# BMPER is a marker of adipose progenitors and adipocytes and a positive modulator of adipogenesis

**DOI:** 10.1038/s42003-023-05011-w

**Published:** 2023-06-13

**Authors:** Jacob D. Garritson, Jiabi Zhang, Alan Achenbach, Maroua Ferhat, Emile Eich, Chris J. Stubben, Paige L. Martinez, Anna R. Ibele, Keren I. Hilgendorf, Sihem Boudina

**Affiliations:** 1 Department of Nutrition and Integrative Physiology, College of Health, University of Utah, Salt Lake City, UT 84112 USA; 2 Bioinformatics Shared Resource, Huntsman Cancer Institute, University of Utah, Salt Lake City, UT USA; 3 Department of Surgery, University of Utah School of Medicine, Salt Lake City, UT USA; 4 Department of Biochemistry, University of Utah, Salt Lake City, UT 84112 USA

**Keywords:** Obesity, Fat metabolism

## Abstract

Autocrine and paracrine signaling regulating adipogenesis in white adipose tissue remains largely unclear. Here we used single-cell RNA-sequencing (RNA-seq) and single nuclei RNA-sequencing (snRNA-seq) to identify markers of adipose progenitor cells (APCs) and adipogenic modulators in visceral adipose tissue (VAT) of humans and mice. Our study confirmed the presence of major cellular clusters in humans and mice and established important sex and diet-specific dissimilarities in cell proportions. Here we show that bone morphogenetic protein (BMP)-binding endothelial regulator (BMPER) is a conserved marker for APCs and adipocytes in VAT in humans and mice. Further, BMPER is highly enriched in lineage negative stromal vascular cells and its expression is significantly higher in visceral compared to subcutaneous APCs in mice. BMPER expression and release peaked by day four post-differentiation in 3T3-L1 preadipocytes. We reveal that BMPER is required for adipogenesis both in 3T3-L1 preadipocytes and in mouse APCs. Together, this study identified BMPER as a positive modulator of adipogenesis.

## Abstract (web summary)

scRNA-seq and snRNA-seq data identified BMPER, as a potential marker of adipogenic adipose progenitor cells in both human and mouse visceral adipose tissue and in vitro studies showed that BMPER is a critical regulator of adipogenesis.

## Introduction

Obesity continues to be a growing public health concern and a high body mass index (BMI) is routinely associated with cardiovascular disease^1^. More specifically, an accumulation of visceral fat has been shown to predict adverse metabolic outcomes^2^ and cardiovascular disease burden^3^.

White adipose tissue (WAT) expands through either an increase in adipocyte size (hypertrophy) or de novo recruitment and differentiation (hyperplasia) of adipose progenitor cells (APCs). Expansion of visceral adipose tissue is believed to be influenced by both intrinsic and environmental factors^4^. Accumulating evidence suggests that functionally distinct subsets of APCs exist within visceral adipose tissue (VAT) of humans and mice. Early work has highlighted the heterogeneity of visceral APCs and their intrinsic adipogenic potential in vitro^5–7^. These initial results have been substantiated by recent single-cell and single-nucleus transcriptomic data^8–10^. While these studies have advanced our understanding of the cellular composition of VAT, the identification and the functional relevance of APC markers remains to be explored.

Here we used single-cell RNA sequencing (scRNA-seq) and publicly available single nuclei RNA sequencing (snRNA-seq) to identify new APC markers and adipogenic modulators in VAT of humans and mice. We also determined the influence of sex and obesity on visceral APCs adipogenic potential in mice. The key findings of the present paper are the existence of sex-specific and diet-specific effects on visceral APC composition in humans and mice and their adipogenic potential in mice. The originality of the present work lies in the finding that APCs and adipocytes in VAT of humans and mice express a conserved marker called BMPER and that this secreted factor is functionally relevant as it is required for adipogenesis.

## Results

### Single-cell RNA-seq recovers all major cell types in VAT of humans and mice that are influenced by obesity

**Fig. 1:**
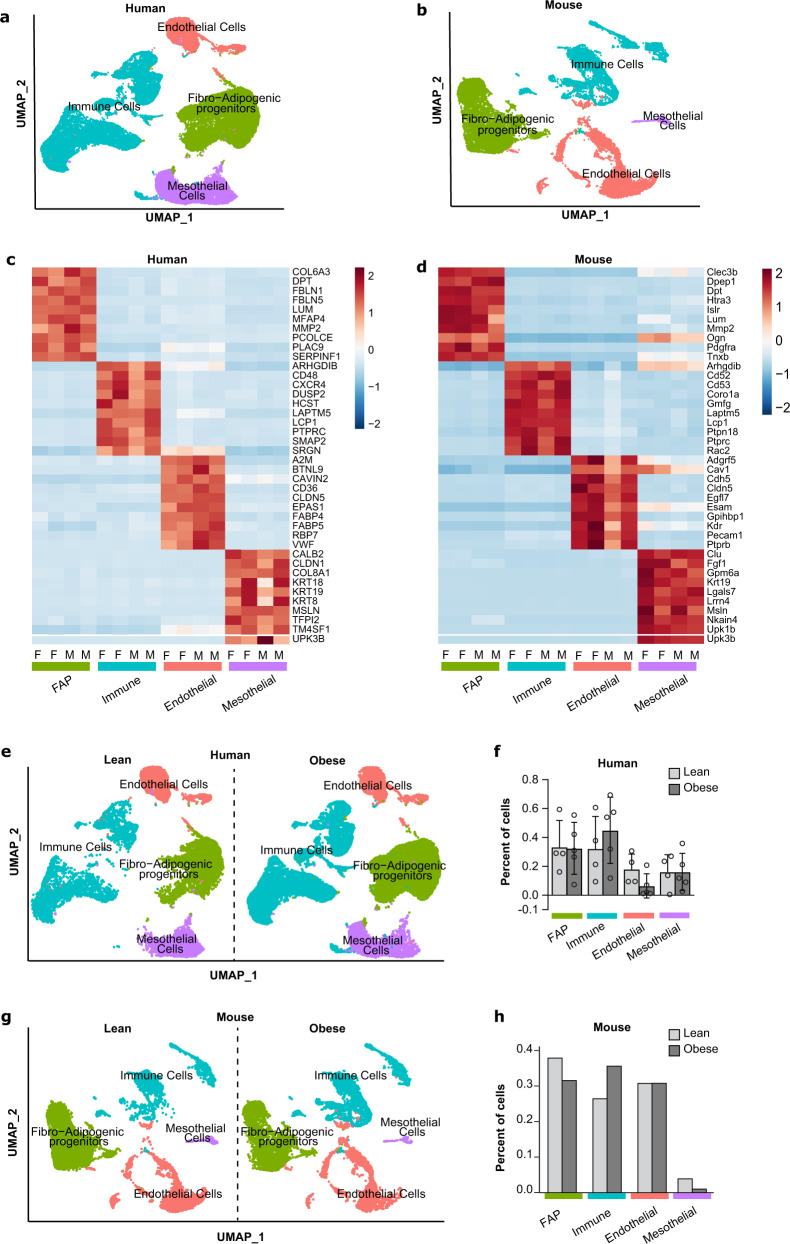
scRNA-seq recovers canonical cell types from visceral adipose tissue (VAT) in humans and mice. **a** Aggregate UMAP of major cell types in the stromal vascular fraction (SVF) of human omental fat. **b** Aggregate UMAP of major cell types in the stromal vascular fraction SVF of mouse perigonadal fat. **c** Heatmap of the top 10 highest expressed genes in each cell type for human females (F) or males (M). **d** Heatmap of the top 10 highest expressed genes in each cell type for mouse females (F) or males (M). **e** UMAPs split by obesity status in humans. **f** Proportion of each cell type in lean or obese human samples. **g** UMAPs split by obesity status in mice. **h** Proportion of each cell type in lean or obese mouse samples. Values are mean ± SD in (**f**). *n* = 4 lean and 5 obese for (**f**) and *n* = 1 pooled sample from *n* = 10 mice fed normal chow diet (lean) and *n* = 1 pooled sample from *n* = 10 mice fed high-fat diet (obese) for (**h**).

To investigate the sex- and obesity/diet-specific differences in the composition of VAT, we performed scRNA-seq on omental fat samples from a small number of lean subjects and subjects with obesity (Table 1). In addition, we performed scRNA-seq on perigonadal white adipose tissue (eWAT) from male (epididymal) and female (periovarian) C57BL/6J mice fed either a normal chow diet (NCD) or high-fat diet (HFD) for 8 weeks. Each sample contained fat tissue pooled from 10 mice, allowing us to analyze sufficient number of cells and while also averaging our analysis across 10 mice. Following rigorous quality-filtering of our scRNA-seq data to remove low-quality cells and cells with a high percentage of mitochondrial DNA, we integrated human and mouse data independent of sex or diet. We identified four major clusters in human and mouse VAT based on differential gene expression (Fig. 1a, b). These clusters are denoted as fibro-adipogenic progenitors (FAPs), immune cells, mesothelial cells, or endothelial cells based on the expression of *PDGFRA*, *PTPRC*, *MSLN*, or *PECAM-1*, respectively (Supplementary Data 1 and 2), and are well separated based on their transcriptional profiles (Fig. 1c, d).

**Table 1:** Human Subject Characteristics.

Subjects	Age	BMI, kg/m^2^
Lean male (*n* = 2)	51.00 ± 7.0	27.57 ± 0.3
Obese male (*n* = 2)	41.00 ± 2.0	46.57 ± 7.5
Lean female (*n* = 2)	60.0 ± 6.0	26.2 ± 1.0
Obese female (*n* = 3)	41.3 ± 7.5	44.2 ± 0.7

Comparison between lean and obese samples in humans, independent of sex, revealed no statistically significant differences in the proportions of cells in each cluster (Fig. 1e, f), which could be related to the small sample size. To further investigate the impact of obesity on the proportions of cells, we analyzed single nuclei RNA-seq of human omental adipose tissue from lean and obese subjects recently published by the Rosen group^10^. As depicted in Supplementary Fig. 1, obesity significantly reduced the proportion of adipose progenitors (referred to in this study as adipose stem and progenitor cells or ASPCs) and significantly increased the proportions of macrophages independently of sex. We observed similar changes to the proportion of immune cells in mouse samples with obesity (Fig. 1g, h and Supplementary Fig. 1). The relative increase in immune cell populations in both humans and mice is consistent with other previous reports^9–12^. As mesothelial cells have been suggested to contribute to VAT formation in mice^13^, we next looked at mesothelial cells proportion. Interestingly, human omental fat contained proportionately more mesothelial cells than mouse perigonadal fat (Fig. 1e–h and Supplementary Fig. 1). One of the recently identified mesothelial cell markers Krt19 was among the genes upregulated in this cell cluster both in humans and mice (Supplementary Data 1 and 2). Whether mesothelial cells contribute to visceral adipocytes in humans is not known, but their contribution to visceral adipocytes in mice has recently been questioned^14^. Taken together, changes in the composition of VAT in obese humans and mice is consistent with previous research with respect to immune cell proportion.

### Sex differences in VAT cellularity in humans and mice

**Fig. 2:**
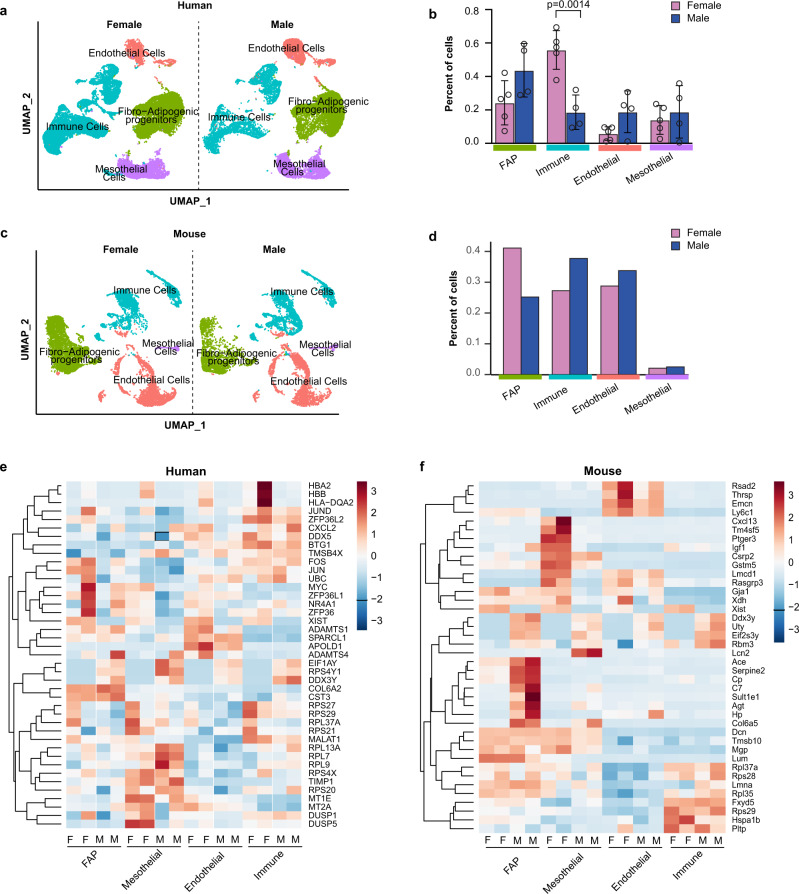
Effects of sex on visceral adipose tissue (VAT) cellular composition in humans and mice. **a** UMAP of major cell types in the SVF of human omental fat samples split by sex. **b** Proportion of each cell type in female or male human samples. **c** UMAP of major cell types in the SVF of mouse perigonadal fat samples split by sex. **d** Proportion of each cell type in female or male mouse samples. **e** Heatmap of the top 10 upregulated genes in each cell type for the female vs. male contrasts in humans. **f** Heatmap of the top 10 upregulated genes in each cell type for the female vs. male contrasts in mice. Values are mean ± SD in (**b**). *n* = 4 females and 5 males for (**b**) and *n* = 1 pooled sample from *n* = 10 male mice and *n* = 1 pooled sample from *n* = 10 female mice for (**d**). **p < 0.005 versus male within the same cell cluster. An unpaired t Test was used to compare the means for each cell cluster.

To uncover sex differences in the cellular composition of VAT, we re-clustered cell types by sex independent of obesity status in each species (Fig. 2a, c). In human VAT, male samples were composed of relatively more FAPs and endothelial cells, whereas samples from females had a significantly higher percentage of immune cells (*p* = 0.0014, Fig. 2b). Interestingly, female mouse VAT samples had proportionately more FAPs and fewer immune cells (Fig. 2d). When we re-clustered cell types by sex and obesity status, similar differences in proportions between sexes were observed (Supplementary Fig. 2). These results were in part supported by the findings from our re-analysis of previously published single nuclei RNA-seq data^10^, demonstrating that female mice had more adipose progenitors and less immune cells when compared to male mice (Supplementary Fig. 1). In contrast, sex had no significant effect on cell proportions in humans in the single nuclei RNA-seq data (Supplementary Fig. 1, unlike what we observed in our single-cell RNA-seq findings. The difference between our human scRNA-seq and the Rosen’s group snRNA-seq results could be related to differences in the sequencing platform or to the ratio of male and female patients.

Next, we performed differential gene expression and pathway analyses between male and female samples from humans and mice (Fig. 2e, f). Comparisons revealed that transcriptional differences between sexes are much more prominent in mice than in humans. For example, male vs. female contrasts in humans found 248, 262, 246, and 204 differentially expressed genes (DEGs) in FAPs, mesothelial, endothelial, and immune cells, respectively (Supplementary Data 3). In comparison, mouse samples had 450, 360, 393, and 332 DEGs (Supplementary Data 4) in the same cell populations highlighting potential limitations in generalizing mouse data across species. Conserved across humans and mice, we observed differential regulation of sex-specific genes between males and females (Supplementary Data 3 and 4). Repression of X inactive specific transcript (*XIST*) and upregulation of ribosomal protein S4 Y-linked 1 (*RPS4Y1*) in males was observed in every cell cluster in both humans and mice (Fig. 2e, f). Interestingly, several sex-specific differences were depicted in the immune cell cluster in humans, with lower hemoglobin subunit (*HBA1* and *HBA2*) expression levels in male vs. female patients (Fig. 2e). In contrast to humans, mice exhibited greater sex differences in the FAP, immune, and mesothelial clusters. Murine male FAPs exhibit a pro-adipogenic/pro-lipogenic transcriptional profile compared to female FAPs evidenced by higher expression of genes known to drive adipogenesis/lipogenesis including sulfotransferase family 1E, member 1 (*Sult1e1*), collagen VI subunit 5a (*Col6a5*), and angiotensin-converting enzyme (*Ace*)^15,16^. In addition, the male mouse immune cluster had higher expression of macrophage-specific transcripts including melanoma protein B (*Gpnmb*) and the lipid-associated macrophage lipid sensor triggering receptor expressed on myeloid cells 2 (*Trem2*). Finally, the male mouse mesothelial cluster expressed less insulin-like growth factor 1 (*Igf1*) and prostaglandin E receptor 3 (*Ptger3*) and more lipocalin 2 (*Lcn2*) transcripts when compared to female mice (Fig. 2f). These results provide new insights into murine-specific transcripts that are differentially regulated by sex and demonstrate that male FAPs express more pro-adipogenic/pro-lipogenic genes compared to female mice.

### Single-cell RNA-seq reveals transcriptionally distinct PDGFRA-expressing cells in VAT of humans and mice

**Fig. 3:**
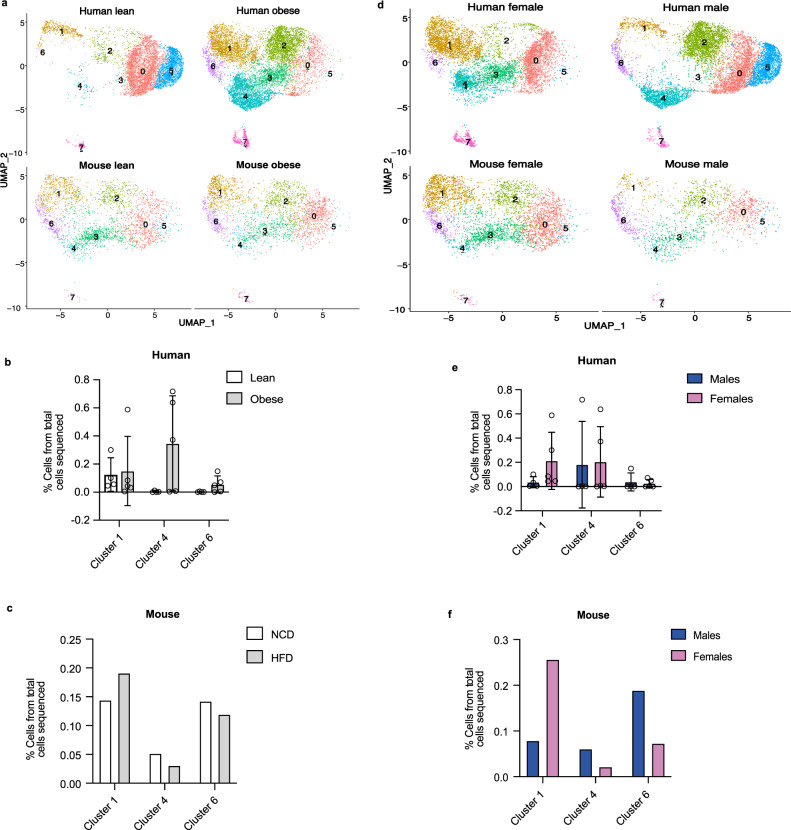
Effects of obesity and sex on FAP clusters in VAT of humans and mice. **a**, **d** UMAPs of FAP clusters in human and mouse separated by obesity status. **b**, **c** The effects of obesity on the proportions of FAP clusters 1, 4, and 6 in humans and mice. **e**, **f** The effects of sex on the proportions of FAP clusters 1, 4, and 6 in humans and mice. Values are mean ± SD. *n* = 4 lean and 5 obese for (**b**); *n* = 4 females and 5 males for (**e**) and *n* = 1 pooled sample from *n* = 10 male mice and *n* = 1 pooled sample from *n* = 10 female mice fed either a normal chow diet (NCD) or a high-fat diet (HFD) for (**c**, **f**).

Since our focus was to identify new VAT APC markers, we re-clustered PDGFRA^+^ FAPs and identified four transcriptionally distinct subpopulations in both humans and mice (Supplementary Fig. 3). First, we clustered human and mouse FAPs without integration and found that the transcriptome of these clusters is very different between humans and mice (Supplementary Data 3 and 4). Thus, we decided to perform a Seurat integration analysis^17^. We selected the FAP clusters in mouse and human, mapped the mouse genes to human homologs from mouse genome informatics (MGI) and then loaded 14,211 common genes into Seurat. We used the SCTransform integration methods (PrepSCTIntegration) to find anchors and combine the two datasets and then ran principal component analysis (PCA) and UMAP clustering with 10 dimensions and 0.4 resolution. As shown in Fig. 3a, we identified seven FAP clusters that are common between humans and mice. We were able to name three clusters based on their gene signature. FAP cluster 1 is enriched with transcripts known to be expressed in uncommitted adipose progenitors including peptidase inhibitor 16 (*PI16*), which was previously shown to mark human and mouse subcutaneous stem cells^18^. This cluster is also enriched in genes related to organ development including smaphorin 3C (*SEMA3C*) and odd-skipped related transcription factor 2 (*OSR2*). FAP cluster 4 represents committed APCs based on the expression of intercellular adhesion molecule 1 (*ICAM1*) and CCAAT enhancer binding protein beta (*CEBPB*). FAP cluster 6 is enriched in genes encoding profibrotic molecules including versican (*VCAN*), microfibril-associated protein 5 (*MFAP5*) and HtrA serine peptidase 3 (*HTRA3*). FAP clusters 0 and 5 may represent committed preadipocytes in different stages of differentiation. FAP cluster 2 represents a stem cell population enriched in adipogenic inhibitors including Thy-1 cell surface antigen (*THY1* or *CD90*) and collagen triple helix repeat containing 1 (*CTHRC1*). Finally, we were not able to fully define FAP clusters 3 and 7, but these cells share gene signatures with immune cells.

Based on the above identification of FAP clusters, we decided to focus on clusters 1, 4, and 6, representing uncommitted APCs, committed APCs, and fibro-progenitors, respectively, and examine the influence of sex and diet on their proportions in human and mice. As shown in Fig. 3a, b, obesity increased the number of committed progenitors and fibro-progenitors in human omental fat, although the difference did not reach statistical significance. Contrary to the results in humans, mice on HFD had more uncommitted APCs compared to committed and fibro-progenitors when compared to their NCD controls (Fig. 3a, c). Moreover, there were no sex-specific differences in the proportions of FAP clusters 1, 4, and 6 that reached significance in humans (Fig. 3d, e). Although qualitative, female mice had more uncommitted APCs and fewer committed APCs and fibro-progenitors when compared to male mice (Fig. 3d, f). Together, these results demonstrate that human omental and mouse perigonadal fat share similar patterns of uncommitted and committed APCs and fibro-progenitors and that obesity induces commitment of APCs in human visceral fat.

### The adipogenic potential of murine visceral adipose progenitors varies by sex and diet

**Fig. 4:**
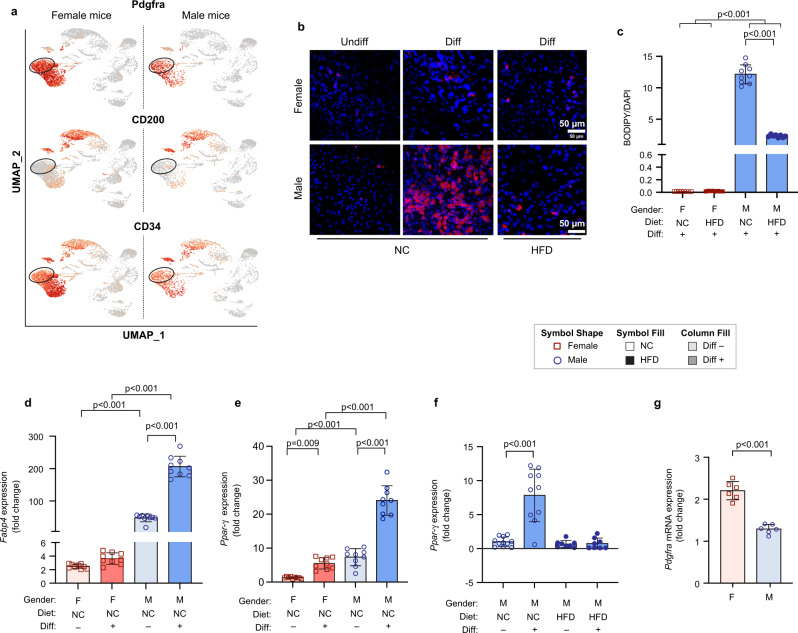
The effect of sex and diet on adipogenic capacity of visceral APCs in mice. **a** Representative UMAPs from male or female mice demonstrating that Pdgfra^+^CD34^low^ cells are CD200^-^. **b** Representative images showing lipid accumulation measured by BODIPY (red) and Dapi (blue) staining following differentiation of CD34^low^CD200^-^ visceral APCs in male or female mice fed either a normal chow (NC) diet or (HFD) for 8 weeks. **c** Quantification of positive BODIPY area normalized to Dapi. **d**, **e** Relative mRNA expression of *Pparγ* and *Fabp4* in undifferentiated (undiff) or differentiated (diff) visceral APCs from female or male mice fed a NC diet. **f** Relative mRNA expression of *Pparγ* in undiff or diff visceral APCs from male mice fed NCD or HFD, respectively. **g** Relative mRNA expression of *Pdgfra* in undifferentiated visceral APCs from female or male mice fed NCD. Values are mean ± SD. *n* = 3 independent experiments. Scale bar in (**b**) is 50 µm.

Motivated by transcriptional differences between male and female visceral APCs (Supplementary Data 3 and 4), we investigated differences in the adipogenic potential of murine APCs from male and female mice in vitro. We previously showed that CD34 can distinguish adipogenic versus non-adipogenic VIS APCs in mice^5^. However, the separation of adipogenic and non-adipogenic APCs with CD34 was not optimal. To characterize new cell surface markers that can better separate adipogenic versus non-adipogenic APCs in VAT of mice, we used our scRNA-seq data and identified CD200 (also known as Ox-2 membrane glycoprotein) as a potential exclusion marker that can further separate CD34^high^ and CD34^low^ APCs in VAT of mice (Fig. 4a). Indeed, a prior study identified CD200 as a marker of visceral stromal stem cells in humans and found that its expression negatively correlates with adipogenic capacity^19^. Thus, we isolated APCs by FACS (Lin^−^, CD34^+^, CD29^+^, Sca1^+^, CD200^-^) from the eWAT of male and peri-ovarian WAT of female mice fed either NCD or HFD for 8 weeks. HFD feeding resulted in significantly higher body weight (BW) in males but not females (Supplementary Fig. 4). Similarly, HFD resulted in worse glucose intolerance in male mice compared to female mice (Supplementary Fig. 4). To investigate sex and diet-specific differences in adipogenesis, we differentiated APCs in the presence of differentiation media (DM). Male APCs from NCD-fed mice were highly adipogenic, while female APCs from NCD-fed mice were refractory to adipogenesis (Fig. 4b, c). In comparison, obesity resulted in a significant decline in the adipogenic potential of male APCs (Fig. 4b, c) and virtually no differentiation in female APCs (Fig. 4b, c). Consistent with lipid accumulation, *Pparg* and *Fabp4* mRNA expression significantly increased with differentiation in NCD male APCs when compared to NCD female APCs (Fig. 4d, e). In line with an obesity-related decline in adipogenic potential of male APCs, *Pparg* mRNA expression was significantly lower in differentiated APCs from obese male mice compared to lean male mice (Fig. 4f). It is important to note that female undifferentiated APCs had much higher *Pdgfra* expression when compared to male mice (Fig. 4g), but these cells were refractory to differentiation in vitro. This is consistent with a recent study that used *Pdgfra* to lineage-trace APCs in vivo and showed that almost no adipocytes were labeled in perigonadal white adipose tissue of female mice^20^.

### Bone morphogenetic protein (BMP) binding endothelial regulator (BMPER) is expressed in human and murine VAT APCs and adipocytes

**Fig. 5:**
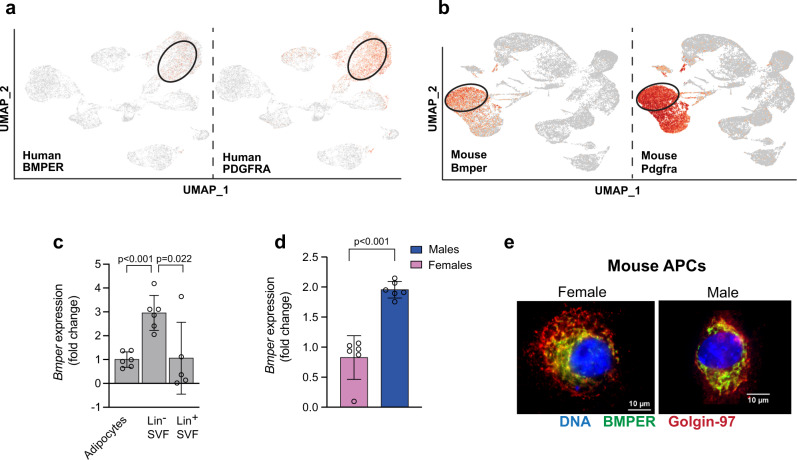
Bone morphogenetic protein (BMP) binding endothelial regulator (BMPER) is highly expressed in visceral APCs in humans and mice. **a** UMAPs of aggregate human omental fat showing BMPER is expressed in the PDGFRA^+^ population. **b** UMAPs of aggregate mouse perigonadal fat showing Bmper is expressed in the Pdgfra^+^ population. **c** Relative mRNA expression of *Bmper* in mature adipocytes, lineage^-^ (Lin^-^), and Lin^+^ SVF isolated from epididymal white adipose tissue of male C57BL6/J control mice. **d** Relative mRNA expression of *Bmper* in undifferentiated visceral APCs from female or male mice. **e** Immunohistochemistry staining for Bmper (green), Golgin-97 (red), or Dapi (blue) in undifferentiated visceral APCs from female or male mice. Values are means ± SD. *n* = two independent experiments in (**c**) and *n* = 6 in (**d**).

As the goal of the present study was to characterize new markers of visceral APCs, we analyzed our scRNA-seq data to identify transcripts that are highly expressed in PDGFRA^+^ APCs both in humans and mice and identified BMPER (Fig. 5a, b). Compared to previous FAP markers such as PDGFRA, PDGFRB, and DPP4, BMPER marked VAT FAPs in humans and mice (Supplementary Fig. 5). As a first step toward understanding the role of BMPER in adipogenesis, we determined the cell type that was most enriched in *Bmper* within the stromal vascular fraction (SVF) in mice. We isolated mature adipocytes, Lin^-^ SVF and Lin^+^ SVF fractions from eWAT of male C57BL/6J mice. Lin^-^ SVF had the highest expression of *Bmper* mRNA when compared to mature adipocytes and Lin^+^ SVF (Fig. 5c). We then examined whether sex influenced *Bmper* mRNA expression in murine visceral APCs. As shown in Fig. 5d, *Bmper* mRNA expression was 2-fold higher in male APCs compared to female APCs (*p* < 0.005). Consistent with Bmper being a secreted protein, co-staining of Bmper and the Golgi marker Golgin-97 was confirmed in male and female undifferentiated visceral APCs (Fig. 5e). Although Bmper was enriched in Lin-SVF, it was not completely absent in mature adipocytes. To confirm whether BMPER expression was also present in adipocytes, we analyzed a recently published snRNA-seq data set^10^. We now reveal that BMPER marks both adipose progenitors as well as mature adipocytes in human omental fat and mouse perigonadal fat independent of sex and obesity (Supplementary Fig. 6).

### BMPER is a positive modulator of adipogenesis in vitro

A previous study showed that Bmper was highly expressed in pericardial adipose tissue stem cells and that its expression was elevated in obesity^21^. However, it remains unknown whether BMPER regulates adipogenesis. As a first step toward understanding the role of BMPER in adipogenesis, we correlated the proportions of BMPER+ cells in the APC cluster from our scRNA-seq on human omental fat with BMI. In addition, we correlated the proportions of BMPER+ ASPCs and adipocytes with BMI in snRNA-seq on human omental fat^10^. The results show that while the proportion of BMPER+ cells in the adipose progenitor cluster negatively correlated with BMI, the proportion of BMPER+ cells in the adipocyte cluster positively correlated with BMI (Supplementary Fig. 7). These results support a pro-adipogenic role for BMPER in VAT in humans.

**Fig. 6:**
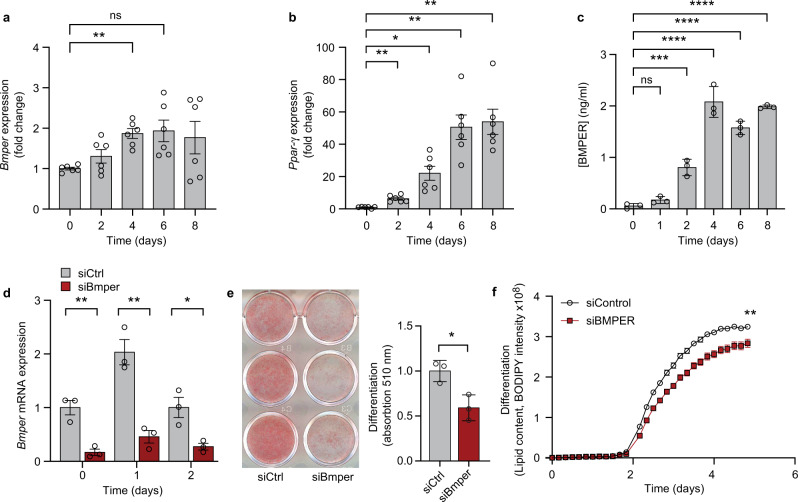
Bmper increases during differentiation of 3T3-L1 preadipocytes and is important for adipogenesis. **a** Relative mRNA expression of *Bmper* in 3T3-L1 cells during different timepoints of differentiation. **b** Relative mRNA expression of *Pparγ* in 3T3-L1 cells during different timepoints of differentiation. **c** Secreted Bmper protein measured during different timepoints of differentiation in 3T3-L1 cells. **d** Relative mRNA expression of *Bmper* following siRNA knockdown in 3T3-L1 cells at different timepoints of differentiation. **e** Oil red O staining and quantification in differentiated 3T3-L1 cells treated with control siRNA or Bmper siRNA knockdown. **f** Lipid content during differentiation in control or Bmper siRNA knockdown measured by BODIPY intensity. Values are means ± SD. *n* = two independent experiments.

Due to the low number of APCs obtainable from mouse perigonadal fat, we first used 3T3-L1 preadipocytes. *Bmper* mRNA expression significantly increased during the differentiation of 3T3-L1 cells in DM (Fig. 6a), suggesting a functional role of Bmper in differentiation of mouse preadipocytes. Increased *Bmper* transcription correlated with the increase in *Pparγ* mRNA (Fig. 6b). Furthermore, Bmper secretion in the media increased during differentiation and peaked at 4 days post-differentiation (Fig. 6c). Next, we examined if lack of Bmper haltered differentiation. 3T3-L1 preadipocytes were transfected with Bmper or scrambled siRNA prior to differentiation and then allowed to differentiate for 8 days. Bmper knockdown was confirmed by reduced *Bmper* mRNA (Fig. 6d). Knockdown of Bmper resulted in significant reduction in lipid accumulation in 3T3-L1 cells cultured in DM (Fig. 6e, f).

**Fig. 7:**
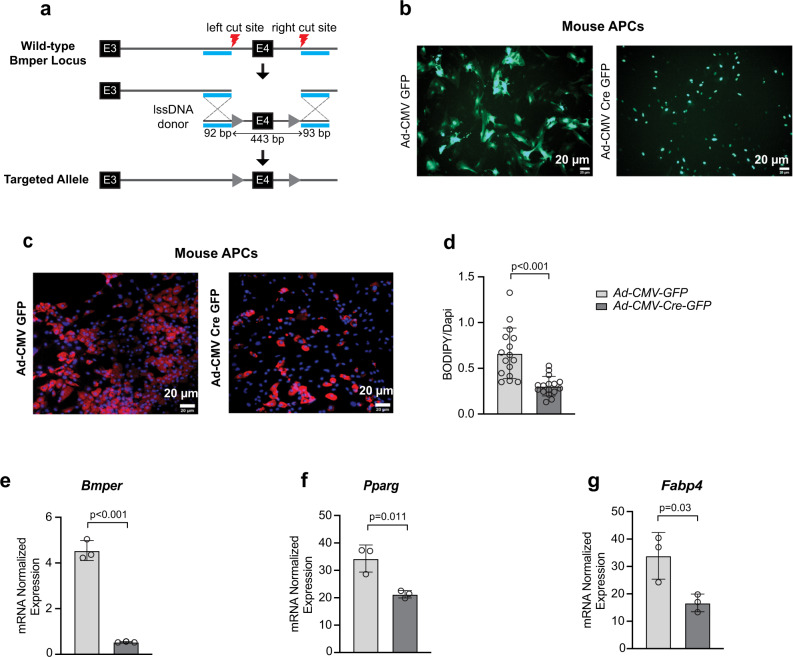
Bmper knockout in mouse primary visceral APCs reduces adipogenesis. **a** Schematic detailing the generation of *Bmper*^*fl/fl*^ mice on a C57BL6/J background using CRISPR/Case 9. Adipose progenitor cells (APCs) were sorted from epididymal white adipose tissue of male *Bmper*^*fl/fl*^ mice. **b** Representative images of green fluorescent protein (GFP) expression in visceral APCs from male *Bmper*^*fl/fl*^ mice infected with adenovirus expressing control GFP (Ad-CM-GFP) or adenovirus expressing Cre-GFP (Ad-CMV-Cre-GFP) prior to differentiation. **c** Representative images showing lipid accumulation in APCs infected with control GFP or Cre-GFP vectors and stained with BODIPY (red) and Dapi (blue) following differentiation. **d** Quantification of positive BODIPY area normalized to Dapi. **e**–**g** Relative mRNA expression of *Bmper*, *Pparγ*, and *Fabp4* in control GFP or Cre GFP following differentiation. Values are mean ± SD. *n* = three independent experiments.

To further validate the role of Bmper in adipogenesis, we generated *Bmper*^*fl/fl*^ mice using CRISPR/Cas9 technology at the University of Utah Mutagenesis Core Facility. To generate a conditional *Bmper* allele, two preassembled RNP complexes were injected to create double-stranded DNA breaks around exon 4 (left/right cut sites). A 628 bp lssDNA donor engineered to contain *LoxP* sites (gray triangles) in the introns around exon 4, flanked by short left and right homology arms (cyan) were co-injected to serve as a DNA repair template. The guide RNAs are designed to direct Cas9-mediated cutting immediately adjacent to each homology arm, which facilitates insertion of the lssDNA donor via HDR. This results in replacement of the wildtype *Bmper* exon 4 with a conditional floxed exon 4 (Fig. 7a). Insertion of the conditional allele were identified *via* simple PCR analyses and founder animals with insertions were sequenced to confirm genotypes.

We then sorted APCs (Lin^-^, CD34^+^, Sca1^+^, Pdgfra^+^, and CD200^-^) from eWAT of male *Bmper*^*fl/fl*^ mice and infected them with either an adenovirus expressing a GFP control or an adenovirus expressing Cre-GFP prior to differentiation. Infection was confirmed by the presence of GFP (Fig. 7b) and resulted in about 90% reduction of *Bmper* mRNA expression (Fig. 7e). Consistent with siRNA knockdown experiments in 3T3-L1 cells, lipid accumulation was significantly decreased in cells treated with Ad-CMV Cre-GFP compared to the control Ad-CMV GFP (Fig. 7c, d). In addition, *Bmper* knockdown significantly reduced *Pparg* and *Fabp4* expression when examined at day 6 post-differentiation (Fig. 7f, g). To verify that the Adeno-Cre did not affect APC differentiation, we transfected visceral APCs isolated from eWAT of male C57BL/6J control mice and showed that this transfection did not affect Bmper expression nor differentiation (Supplementary Fig. 8). To our knowledge, this is the first report that identifies Bmper as a potential APC marker enriched in visceral fat and a positive regulator of adipogenesis.

## Discussion

We employed scRNA-seq to characterize the transcriptome of male and female human and mouse VAT and studied the effect of obesity and sex on the cellular composition of omental fat in humans and perigonadal fat in mice. In addition, this cellular atlas allowed us to characterize additional markers for visceral APCs and to identify a regulator of adipogenesis (BMPER).

We show that the composition of SVF in VAT varies by sex within species. That is, human females appear to have a higher proportion of immune cells and lower proportion of FAPs, while female mice tend to have a lower proportion of immune cells and higher proportion of FAPs. Additional studies are needed to confirm this observation as previous scRNA-seq or snRNA-seq did not compare immune cell proportions between sexes^10,22^. While the increase in immune cell infiltration observed with obesity in mice is consistent with previous studies^9, 11, 12^, little work has been done to directly compare sex and diet-induced changes to VAT cellular composition in humans and mice, except for a recent study by the Rosen lab, which also showed that immune cell infiltration is more prominent in HFD-fed male mice^10^. Comparisons between sexes in our study revealed that canonical cell types in the VAT SVF are much more transcriptionally distinct in mice compared to humans. This could be due to the limited number of patient samples that were included in our study (4 males and 5 females). In mice, male FAPs expressed higher levels of *Sult1e*1 (encoding estrogen sulfotransferase or EST) compared to females. This gene is highly expressed in male white adipose tissue and plays a role in estrogen inactivation^23^. Overexpression of EST in human adipose stem cells promoted adipogenesis^15^. Similarly, *Col6a5*, which was previously shown to be induced by dihydrotestosterone, is higher in male mice compared to female mice within the FAP cluster. Deletion of this gene in 3T3-L1 preadipocytes prevented lipid accumulation^16^. Enrichment of these genes in male mice FAPs may suggest that these cells are more adipogenic/lipogenic when compared to female mice FAPs. Our pathway analysis further confirmed elevation of the adipogenic pathway in male versus female mice FAPs (Supplementary Data 5 and 6). Contrary to FAPs, the mouse endothelial cell cluster did not reveal significant sex differences. However, the immune clusters were transcriptionally distinct between male and female mice. The difference seems to affect the macrophage subtype based on differentially expressed macrophage-specific transcripts. Although we did not perform sub-clustering of the immune cluster, we observed an increase in *Lgals3*, *Gpnmb*, *Capg*, *Trem2*, *Anxa1*, and *Mmp12* transcripts in male mice compared to female mice. These transcripts characterize lipid-associated macrophages, previously identified in male gonadal fat of HFD-fed mice^11^. Similarly, *Gpnmb* expression is increased in adipose tissue of male mice during obesity and has been involved in macrophage activation^24^. These changes further confirm the accepted notion that male mice are more prone to accumulate macrophages in VAT during obesity compared to female mice^10^.

We demonstrated that mice have proportionately fewer mesothelial cells (Fig. 1), which is consistent with previous work^10^. Despite low mesothelial cell number, male mice display a distinct gene signature compared to female mice. Notably, male mice expressed less prostaglandin E receptor 3 (*Ptger3*) transcript compared to females. Deletion of this gene caused abnormal lipid distribution and resulted in ectopic lipid accumulation and insulin resistance only in male mice^25^. Male mice mesothelial cells had a 2-fold increase in lipocalin 2 (*Lcn2)* compared to females, and this gene has been previously linked to sex-specific differences in adipose tissue metabolic function^26^. Taken together, our study reveals important sex-specific differences in transcriptional profiles of VAT SVFs that are more pronounced in mice and are consistent with a pro-adipogenic/pro-inflammatory transcriptional profile in male versus female mice.

Unbiased re-clustering of PDGFRA^+^ cells identified four transcriptionally distinct FAP populations in mice and humans. Our study is consistent with previous scRNA-seq or snRNA-seq studies in mice, which also identified four main FAP clusters in epididymal fat of male mice^6, 8, 9, 27^. To our surprise, the identified FAP clusters had little overlap in gene expression (Supplementary Fig. 3). Thus, we performed an integration analysis, which allowed us to identify shared cell states between human and mouse FAPs. This analysis had the advantage of identifying similar cell states between human and mouse adipose progenitors. We identified three FAP clusters: uncommitted progenitors (FAP1), committed progenitors (FAP4), and fibro-progenitors (FAP6). Due to the relatively small number of patients included in this study, we were unable to detect significant differences in the proportions of these clusters based on obesity or sex. Future studies including more patients are underway to study the effect of diet and sex on FAP clusters in humans.

Another important finding from the current study is the effect of diet and sex on the adipogenic potential of visceral APCs. We found that APCs from male mice fed NCD are highly adipogenic, while APCs from female mice fed the same diet are refractory to adipogenesis in vitro. In addition, HFD reduced adipogenesis of VAT APCs in male mice. The effect of obesity on adipogenesis has been examined in the subcutaneous fat of men, and it was shown that adipogenesis is negatively correlated with BMI and adipocyte size^28^. This effect is potentially mediated through a tumor necrosis factor (TNF) α-dependent inhibition of PPARα suggesting that obesity-related inflammation may contribute to reductions in the adipogenic potential of APCs^29^. Furthermore, it has recently been reported that nuclear estrogen receptor α (Erα) and wingless-integrated 1 (Wnt1) inhibit adipogenesis in visceral APCs and 3T3-L1 cells^30^. In addition, Ahluwalia and colleagues observed a direct binding of estrogen receptor α to the Pparγ promoter and a recruitment of transcriptional repressors of adipogenesis such as Gata3 and Wnt when 3T3-L1 or adipose stem cells were differentiated in the presence of estrogen^30^. These results and the results from the current study suggest that estrogen-dependent transcriptional and epigenetic modifications in female visceral APCs may reduce the adipogenic potential of these cells in vitro.

The main goal of this study was to discover new markers for visceral APCs to improve their purification. Our scRNA-seq data identified BMP endothelial cell precursor-derived regulator (BMPER), also known as Crossveinless-2 (CV2), as a potential marker of adipogenic APCs in both human and mouse VAT. To confirm our finding, we analyzed snRNA-seq of human omental fat and mouse perigonadal fat from the Rosen’s group^10^ and showed that BMPER marked both APCs (named ASPCs in this study) and mature adipocytes (as the nuclei of these cells were captured).

BMPER was first identified in *Drosophila* to be required for the formation of wing crossveins^31^. BMPER was then described as a secreted protein that modulates Bmp4 signaling in endothelial precursor cells^32^. Relevant to this study, knockout of Bmper in endothelial cells resulted in hyperinsulinemia and glucose intolerance^33^, but this study did not examine the role of BMPER in adipogenesis. As BMP4 is known to induce adipogenesis^34^ and BMPER has been shown to modulate BMP action in endothelial cells and during organogenesis^32, 35–38^, we hypothesized that BMPER may modulate adipogenesis in APCs. Indeed, Bmper knockdown experiments in 3T3-L1 cells reduced lipid accumulation. In addition, knockout of Bmper in primary mouse visceral APCs resulted in decreased lipid accumulation and adipogenic gene transcription following differentiation. These findings support that BMPER is a positive regulator of adipogenesis.

The mechanisms by which BMPER regulates adipogenesis are currently not known but our findings may suggest that BMPER is needed for the initiation of differentiation as its deletion prior to exposure to DM reduced adipogenesis. As BMPER was previously shown to impact migration but not proliferation of pericardial stem cells^21^, it is possible that BMPER impacts clonal expansion and migration early during the commitment phase of adipogenesis. Furthermore, owing to its BMP modulation effects^39^ and because BMPs (namely BMP4) have previously been shown to promote commitment of human adipose stem cells to the adipogenic lineage^40^, we propose that BMPER modulates BMP signaling in APCs to facilitate their commitment to the adipogenic lineage. In support of this idea, we observed a decrease in *PPARγ* and *Fabp4* mRNA expression in Bmper deleted cells, suggesting Bmper acts upstream of these adipogenic regulators and controls their transcription by modulating the concentration of BMPs. As BMPER is highly enriched in adipocytes (as shown in the snRNA-seq), it is also possible that this factor may modulate lipid accumulation through its insulin sensitizing effect^33^. Further work is needed to identify the mechanism by which BMPER modulates adipogenesis.

We acknowledge that the present study has several limitations including a small number of patients and that interpatient variability makes it difficult to reach statistical significance. However, compared to available sequencing data in human omental fat, this study has very well-matched subjects in terms of age and BMI. In addition, we recognize that female subjects were menopausal, and this may have affected the cellular composition of omental fat. Although we identified BMPER as a marker for APCs based on scRNA-seq, we now show that it is also expressed in adipocytes using publicly available single nuclei sequencing^10^. We also acknowledge that differences in depot locations (omental in humans versus gonadal in mice) and sex hormones may have influenced the changes we observed in the proportions of cell clusters in both humans and mice. Finally, this study focused primarily on the VAT depot, as the original intent was to identify new VAT APC markers.

## Methods

### Human subject characteristics

Visceral (greater omental) adipose tissue samples were obtained in consented patients for our University of Utah Institutional Review Board (IRB)-approved obesity biorepository at the time of laparoscopic bariatric surgery (obesity group) or laparoscopic abdominal surgery for non-cancerous, non-infected general surgery indications (lean group). Patients were fasted for a minimum of 7 h prior to surgery, and bariatric patients were given a high-protein very low-calorie diet for 4 weeks prior to surgery. Patients were excluded from the repository if they had active malignancy, active infection, or an inflammatory general surgical issue such as appendicitis or cholecystitis at the time of surgery. The number of patients included in this study is 9 (2 lean males, 2 obese males, 2 lean females, and 3 obese females). Patient’s characteristics is provided in Table 1. In addition, we used publicly available single nuclei RNA-seq data (GSE176171) set and analyzed only omental fat samples from 2 lean females, 2 obese females, 1 lean male and 2 obese males. The characteristics of the patients included for the single nuclei RNA-seq analysis is shown in Supplementary Table 1.

### Animals and diet

All animals were treated in accordance with the University of Utah, Institutional Animal Care and Use Committee guidelines and policies. We comply will all relevant ethical regulations for animal testing.

8-week-old male or female C57BL/6J mice were fed one of two diets ad libitum for 8 weeks: a normal chow diet (NCD; Research Diets Inc.) containing 10% fat, 70% carbohydrate, and 20% protein or a high-fat diet (HFD; Envigo) containing 60% fat, 20% carbohydrate, and 20% protein (given as percentages of total kilocalorie content).

In order to generate a conditional Bmper allele, two preassembled ribonucleoprotein (RNP) complexes were injected to create double-stranded DNA breaks around exon 4 (left/right cut sites, intron 3 gRNA: 5’-GGGAAAGAAAACGTTGCCAA-3’, intron 4 gRNA: 5’-CCAAGCCTTGCTTGGTTAGT-3’). A 628 bp lssDNA donor (IDT megamer) engineered to contain LoxP sites (Fig. 7a gray triangles) in the introns flanking exon 4, flanked by short left and right homology arms (Fig. 7a cyan) was co-injected to serve as a DNA repair template.

### Glucose and insulin tolerance tests (GTT and ITT)

Mice were fasted 4 h prior to ITT or 5 h prior to GTT, starting in the morning (8 am). Baseline fasting blood glucose was measured from venous blood via a small tail tip cut before insulin or glucose administration. Insulin (1 U/kg body weight for ITT) or glucose (2 g/kg body weight for GTT) was injected interperitoneally. Blood glucose values were obtained at 15, 30, 60, 90, and 120 min after baseline using a glucose meter.

### Body composition measurement

Body composition was determined using a Bruker Minispec whole body composition analyzer (Bruker Optics, Inc.) based on nuclear magnetic resonance. Animals were placed in a clear plastic cylinder and immobilized by insertion of a plastic plunger. The tube was lowered into the analyzer to assess lean mass, fat mass, and fluid mass.

### Preparation of stromal vascular fraction (SVF) for single-cell sequencing

Human and mouse SVFs were isolated as described below. Briefly, perigonadal fat from male and female mice was dissected and dissociated by collagenase digestion for 30 min. Following enzymatic digestion, SVFs were incubated in red blood cells lysis buffer for 2 min, washed with HBSS, resuspended in PBS + 0.04% BSA at a concentration of 1200 cells/µl, and submitted for sequencing by 10X Genomics.

### Sequencing and data analysis

Sequencing libraries were chemically denatured and applied to an Illumina NovaSeq flow cell using the NovaSeq XP workflow (20043131). Following transfer of the flowcell to an Illumina NovaSeq 6000 instrument, a 150 × 150 cycle paired-end sequence run was performed using a NovaSeq 6000 S4 reagent Kit v1.5 (20028312).

The Fastq files were aligned to the GRCh38 human or GRCm38 mouse reference from 10X genomics (version 3.0.0) using cellranger count version 3.1.0 to create quality control metrics, Loupe Browser files, and filtered gene barcode matrices. The gene barcode matrices from the nine human samples were loaded into Seurat version 4.0.1 in R and merged into a single matrix^41^. Cells with more than 15% mitochondrial reads, less than 300 features, or greater than 6000 features were removed and counts were normalized using the SCTransform method^42^. Nearest neighbors and clusters were identified using 15 dimensions and a 0.5 resolution and UMAP plots were generated for visualizations. Four main cell types were identified by plotting expression of marker genes like PDGFRA, PTPRC, MSLN, and PECAM-1 and by assigning cell types using a nearest neighbor classifier in the SingleR package^43^. Cluster markers and significant genes between lean male vs. lean female, obese male vs lean male, obese female vs lean female, obese vs lean, and male vs female within the four main cell types were identified using the default Wilcoxon Rank Sum test. In addition, the same contrasts were analyzed in FAP cell types using pseudobulking methods. The significant genes were analyzed using the clusterProfiler package to identify over-represented gene sets and WikiPathways using a Fisher’s Exact Test^44^. The four mouse samples were analyzed in a similar manner except that a 10% cutoff for mitochondrial reads was used.

### Immunocytochemistry

Cells were grown on 12 mm round coverslips and fixed with 4% paraformaldehyde (433689M, AlfaAesar) in PBS at room temperature for 10 min. Samples were blocked with 5% normal donkey serum (017-000-121, Jackson ImmunoResearch) in immunofluorescence (IF) buffer (3% BSA and 0.1% NP-40 in PBS) at room temperature for 30 min. Samples were incubated with primary antibody in IF buffer at room temperature for 1 h, followed by 5 washes with IF buffer. Samples were incubated with fluorescent-labeled secondary antibody at room temperature for 30 min, followed by a 5 min incubation with 4’,6-diamidino-2-phenylindole (DAPI) in PBS at room temperature for 5 min and 5 washes with IF buffer. Coverslips were mounted with Fluoromount-G (0100-01, SouthernBiotech) onto glass slides followed by image acquisition. The following primary antibodies were used: BMPER (AB73900, 1:150), Golgin-97 (A-21270, 1:200).

### 3T3-L1 culture, differentiation, lipid staining, and transfection

3T3-L1 cells were purchased from ATCC and grown to confluency in DMEM containing 10% bovine calf serum, followed by another 2 days at confluency in DMEM containing 10% bovine calf serum. Adipogenesis was then induced using DMEM containing 10% FBS and differentiation cocktail consisting of 1 μg/ml insulin, 1 μM Dex, and 0.5 mM IBMX. After 2 days of differentiation cocktail, media was changed to DMEM containing 10% FBS and 1 μg/ml insulin. Maintenance media was changed every 2–3 days for a total differentiation time of 4–8 days.

Adipogenesis was quantified using Oil Red O staining. Cells were fixed in 4% PFA/PBS for 10 min at room temperature, followed by 3 rinses in PBS. Samples were incubated in 60% isopropanol for 5 min at room temperature and then allowed to dry completely. Samples were then incubated in freshly diluted 60% Oil Red O staining solution in water (stock: 0.5% Oil Red O (Sigma, 00625) in isopropanol) for 20 min at room temperature, followed by 3 rinses in water. Samples were allowed to dry completely and imaged. For quantification, Oil Red O was extracted by incubating dried samples stained on the same day in 100% isopropanol for 5 min at room temperature and absorbance was measured at 510 nm.

For the kinetic quantification of adipogenesis, 3T3-L1 cells were differentiated as described above and supplemented with 200 nM BODIPY 493/503, and green fluorescence images were acquired every 2 h using default IncuCyte setting. Total green fluorescence intensity was determined from a green fluorescent mask generated by the IncuCyte Zoom Analysis Software.

For knockdown experiments using siRNA, 3T3-L1 cells were transfected with 25 nM siRNA using DharmaFECT Reagent 1. Media was changed after 24 h, and differentiation was initiated 96 h post-transfection.

### Quantification of secreted BMPER by ELISA

3T3-L1 cells were differentiated as described above. Media was collected at the indicated timepoints of 3T3-L1 differentiation. The concentration of BMPER in the media was measured by enzyme-linked immunosorbent assay (ELISA). The ELISA was performed according to the manufacturer’s instructions (ABIN649186).

### Adipose progenitor cell isolation and differentiation

SVFs were isolated from perigonadal white adipose tissue of male and female mice fed NCD or HFD as previously described^5^. Briefly, SVFs were incubated in red blood cell lysis buffer for 2 min, suspended in HBSS, and labeled with antibodies against surface markers identified to mark APCs. In addition to the surface markers previously used to identify APCs by our lab^5^, we identified CD200 as an additional marker to purify APCs (Lin^-^ CD200^-^ Sca1^+^ CD34^low^). APCs were then separated by fluorescence-activated cell sorting (FACS) under sterile conditions (Supplementary Fig. 9) and seeded at 5 × 10^4^/cm^2^ in progenitor media (PM) consisting of DMEMF-12 supplemented with 10% FBS, 100 U/ml penicillin, 100 μg/ml streptomycin, and 10 ng/ml bFGF.

Sorted APCs were cultured in growth media (GM) (DMEMF-12, 10% FBS, 100 U/ml penicillin, 100 μg/ml streptomycin) until >75% confluency (3–4 days) and then exposed to differentiation media (DM) containing 1 μg/ml insulin, 0.25 mg/ml dexamethasone and 0.5 mmol/L isobutylmethylxanthine (IBMX) in DMEM-F12 with 10% FBS and 1% penicillin/streptomycin. Cells were cultured in DM for 3 days, then switched to GM and cultured for an additional 3 days before extracting RNA or staining neutral lipids with BODIPY.

### BODIPY staining

Cells were fixed in 4% paraformaldehyde for 15 min at room temperature and then rinsed three times with PBS. Cells were stained with BODIPY (10 mg/ml) for 1 h at room temperature. Wells were washed three times with PBS and then mounted with ProLong Gold Antifade Mountant with DAPI. The positive area of BODIPY was normalized to DAPI using ImageJ.

### Primary APCs transfection and differentiation

Visceral APCs were isolated as described above from male *Bmper*^*fl/fl*^ mice and infected with either adenovirus expressing GFP (Ad CMV eGFP; FVQ002, Kerafast) or adenovirus expressing Cre-GFP (Ad CMV Cre- RSV GFP; FVQ005, Kerafast) using a PFU of 6 × 10^8^/well for 48 h. Following infection, cells were differentiated as described above. Lipid accumulation was quantified using BODIPY. *Bmper*, *Pparγ*, and *Fabp4* mRNA expression were quantified by qPCR.

### qPCR analysis

Total RNA was isolated with TRIzol (Life Technologies, Grand Island, NY, USA) according to the manufacturer’s instructions. For the quantitative PCR, the final reaction volume was 12 µl and included specific primers, 10 ng of cDNA, and the SYBR green master mix (Life Technologies, Grand Island, NY, USA). The quantitative PCR assays were run on an ABI Prism 7900HT real-time PCR machine (Applied Biosystems, USA). Normalization was performed using ribosomal protein L13 (Rpl13) RNA. Quantification was performed using the comparative ∆Ct method.

### Statistics and reproducibility

The data are presented as mean ± standard deviation of the mean. All statistical analyses were performed using GraphPad Prism software version 9. The value of *p* < 0.05 was considered statistically significant for all the experiments. Significant differences between two groups and within the same cell cluster were evaluated using an unpaired two-tailed *t*-test. Two-way ANOVA was used to determine significance among means with two independent variables. If significance was obtained, a Tukey’s post hoc test was used to identify the location of the differences. Significance is indicated on each graph.

Sample size for single-cell RNA sequencing included 9 patients (2 lean males, 2 obese males, 2 lean females, and 3 obese females). To add rigor, we also used publicly available single nuclei RNA sequencing data set (GSE176171) and analyzed only omental fat samples from 7 patients (2 lean females, 2 obese females, 1 lean male, and 2 obese males). For mice single-cell RNA sequencing, we pooled perigonadal fat from 10 mice for each sample. We used four samples: one NCD male, one HFD male, one NCD female, and one HFD female. In addition, we used publicly available single nuclei RNA sequencing data set (GSE176171) and analyzed only perigonadal fat samples from 12 mice (2 NCD females, 2 HFD females, 3 NCD males, and 5 HFD males).

### Reporting summary

Further information on research design is available in the Nature Portfolio Reporting Summary linked to this article.

## Supplementary information

Supplemental Material

Description of Additional Supplementary Files

Supplementary Data 1

Supplementary Data 2

Supplementary Data 3

Supplementary Data 4

Supplementary Data 5

Supplementary Data 6

Supplementary Data 7

Reporting Summary

## Data Availability

All the data supporting this work is available in the paper and in the supplementary material. The single-cell RNA sequencing data is deposited in Gene Expression Omnibus (GEO) with the accession number GSE214982. Single nuclei RNA sequencing data is publicly available and deposited in GEO with the accession number GSE176171. All raw datasets are available in the source data table provided as a supplementary material. Source data can be found in the file named Supplementary Data 7. All other data are available from the corresponding author on reasonable request.
