# Supplementary material for: BMPER is a marker of adipose progenitors and adipocytes and a positive modulator of adipogenesis
Source: Commun Biol. 2023 Jun 13;6:638. doi: 10.1038/s42003-023-05011-w (PMC10264349; doi:10.1038/s42003-023-05011-w)
Supplement: Supplementary file 1 — Supplemental Material [file 42003_2023_5011_MOESM1_ESM.pdf]

BMPER is a marker of adipose progenitors and adipocytes and a positive modulator of adipogenesis

Jacob D. Garritson, Jiabi Zhang, Alan Achenbach, Maroua Ferhat, Emile Eich, Chris J. Stubben, Paige L. Martinez, Anna R. Ibele, Keren Hilgendorf and Sihem Boudina

Supplemental Material

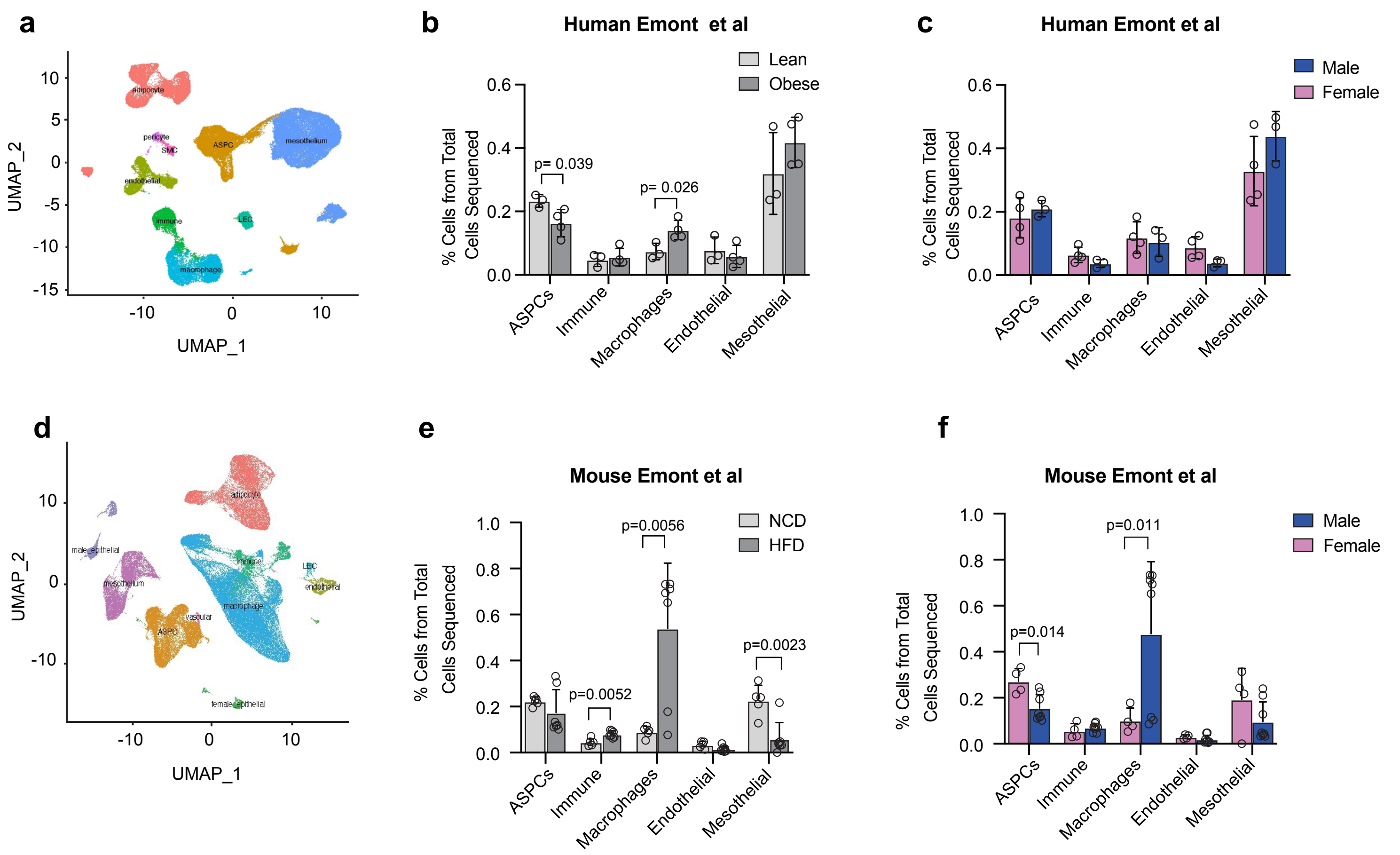
a
UMAP_2
UMAP_1
adipocyte
pericyte
SMC
ASPC
mesothelium
endothelial
immune
LEC
macrophage
b
Human Emont et al
Lean
Obese
% Cells from Total Cells Sequenced
p= 0.039
p= 0.026
ASPCs
Immune
Macrophages
Endothelial
Mesothelial
c
Human Emont et al
Male
Female
% Cells from Total Cells Sequenced
ASPCs
Immune
Macrophages
Endothelial
Mesothelial
d
UMAP_2
UMAP_1
adipocyte
male_epithelial
mesothelium
immune
LEC
endothelial
macrophage
vascular
ASPC
female_epithelial
e
Mouse Emont et al
NCD
HFD
% Cells from Total Cells Sequenced
p=0.0056
p=0.0052
p=0.0023
ASPCs
Immune
Macrophages
Endothelial
Mesothelial
f
Mouse Emont et al
Male
Female
% Cells from Total Cells Sequenced
p=0.011
p=0.014
ASPCs
Immune
Macrophages
Endothelial
Mesothelial

Supplementary Figure 1: Re-analysis of single nuclei RNA sequencing (snRNA-seq) data published by Emont MP et al., Nature 2022 (GSE176171). A. Aggregate UMAP of major cell types in the stromal vascular fraction (SVF) of human omental fat from 7 patients (1 lean male, 2 obese males, 2 lean females and 2 obese females). **B**. Aggregate UMAP of major cell types in the stromal vascular fraction (SVF) of mouse from epididymal fat samples from 5 normal chow diet (NCD) and 5 high fat diet (HFD) fed male mice and periovarian fat samples from 2 NCD and 2 HFD female mice respectively. **C**. Proportion of each cell type in lean or obese male and female subjects. **D**. Proportion of each cell type in male or female lean and obese subjects. **E**. Proportion of each cell type in male or female NCD and HFD mice. **F**. Proportion of each cell type male or female mice fed NCD and HFD. Values are mean ± SD. *p<0.05; **p<0.005 versus lean or versus male assessed by a *t* Test in each cell cluster.

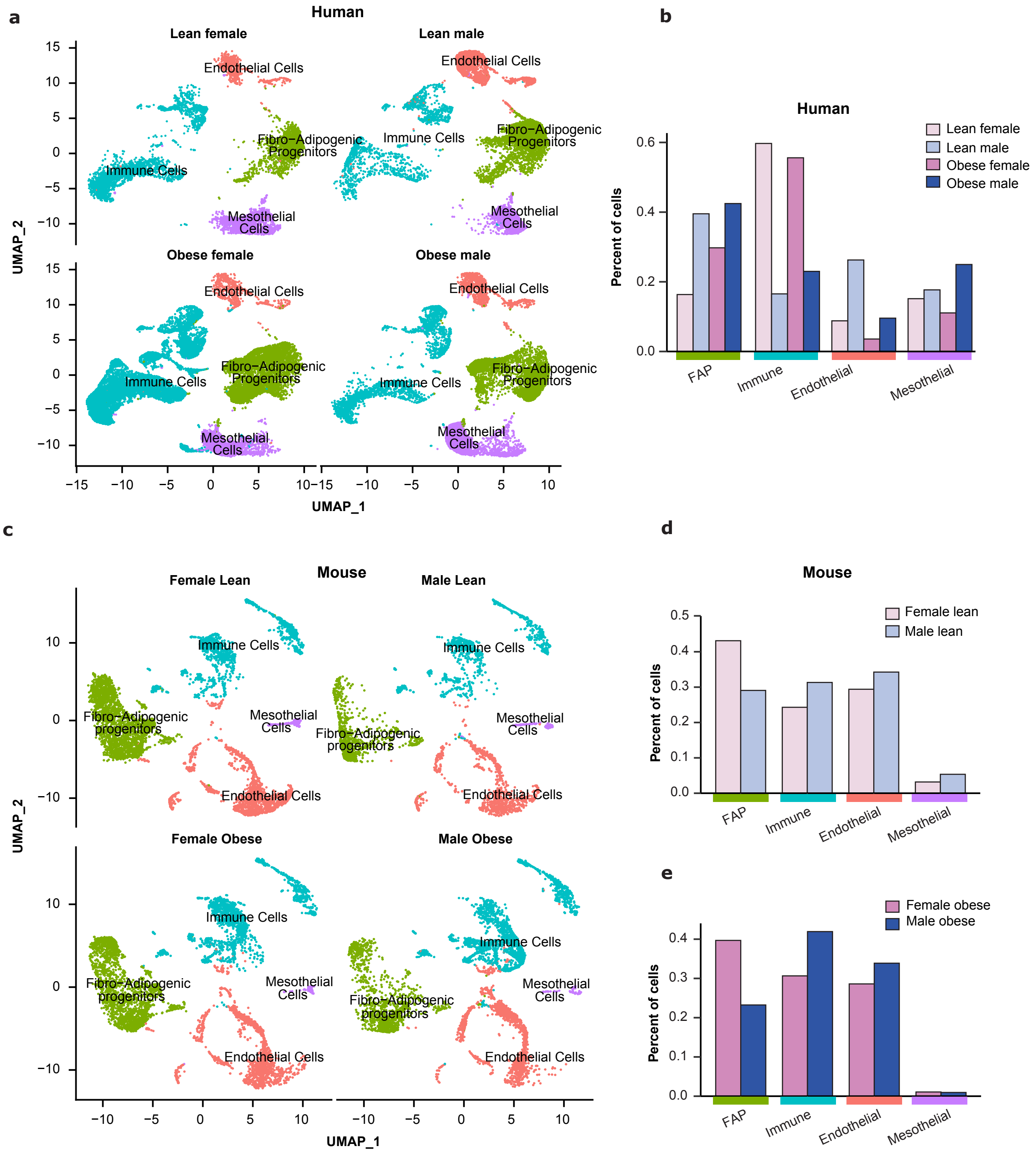
a
Human
Lean female
Lean male
Obese female
Obese male
Endothelial Cells
Fibro−Adipogenic Progenitors
Immune Cells
Mesothelial Cells
UMAP_2
UMAP_1
b
Human
Lean female
Lean male
Obese female
Obese male
Percent of cells
FAP
Immune
Endothelial
Mesothelial
c
Mouse
Female Lean
Male Lean
Female Obese
Male Obese
Immune Cells
Fibro−Adipogenic progenitors
Mesothelial Cells
Endothelial Cells
UMAP_2
UMAP_1
d
Mouse
Female lean
Male lean
Percent of cells
FAP
Immune
Endothelial
Mesothelial
e
Female obese
Male obese
Percent of cells
FAP
Immune
Endothelial
Mesothelial

Supplementary Figure 2: Clustering of male and female visceral SVF cell clusters separated by obesity status.

A. UMAP of major cell types in the SVF of human omental fat samples from male and female patients separated by obesity status. **B**. Proportion of each cell type in female or male lean or obese human samples. **C**. UMAP of major cell types in the SVF of male and female mouse perigonadal fat samples separated by diet. **D** and **E**. Proportion of each cell type in female or male mouse samples separated by diet.

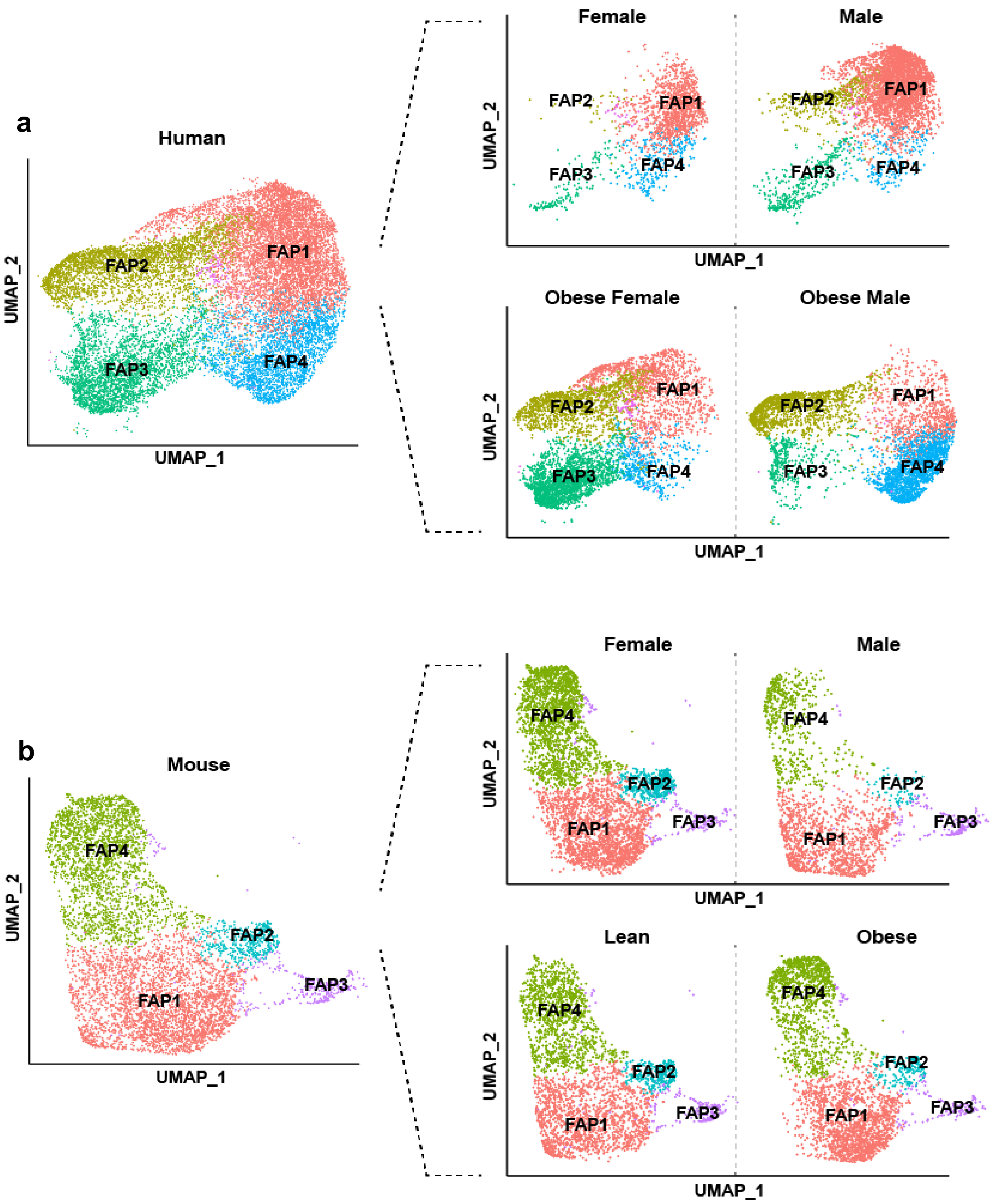

a
Human
FAP1
FAP2
FAP3
FAP4
UMAP_1
UMAP_2
Female
Male
Obese Female
Obese Male
b
Mouse
Lean
Obese

Supplementary Figure 3: Heterogeneity of fibro-adipogenic progenitors (FAPs) in VAT of humans and mice.

A. UMAP of sub-clustering of aggregate male and female FAPs in humans; split by sex and obesity status. **B**. UMAP of sub-clustering of aggregate male and female FAPs in mice; split by sex and obesity status.

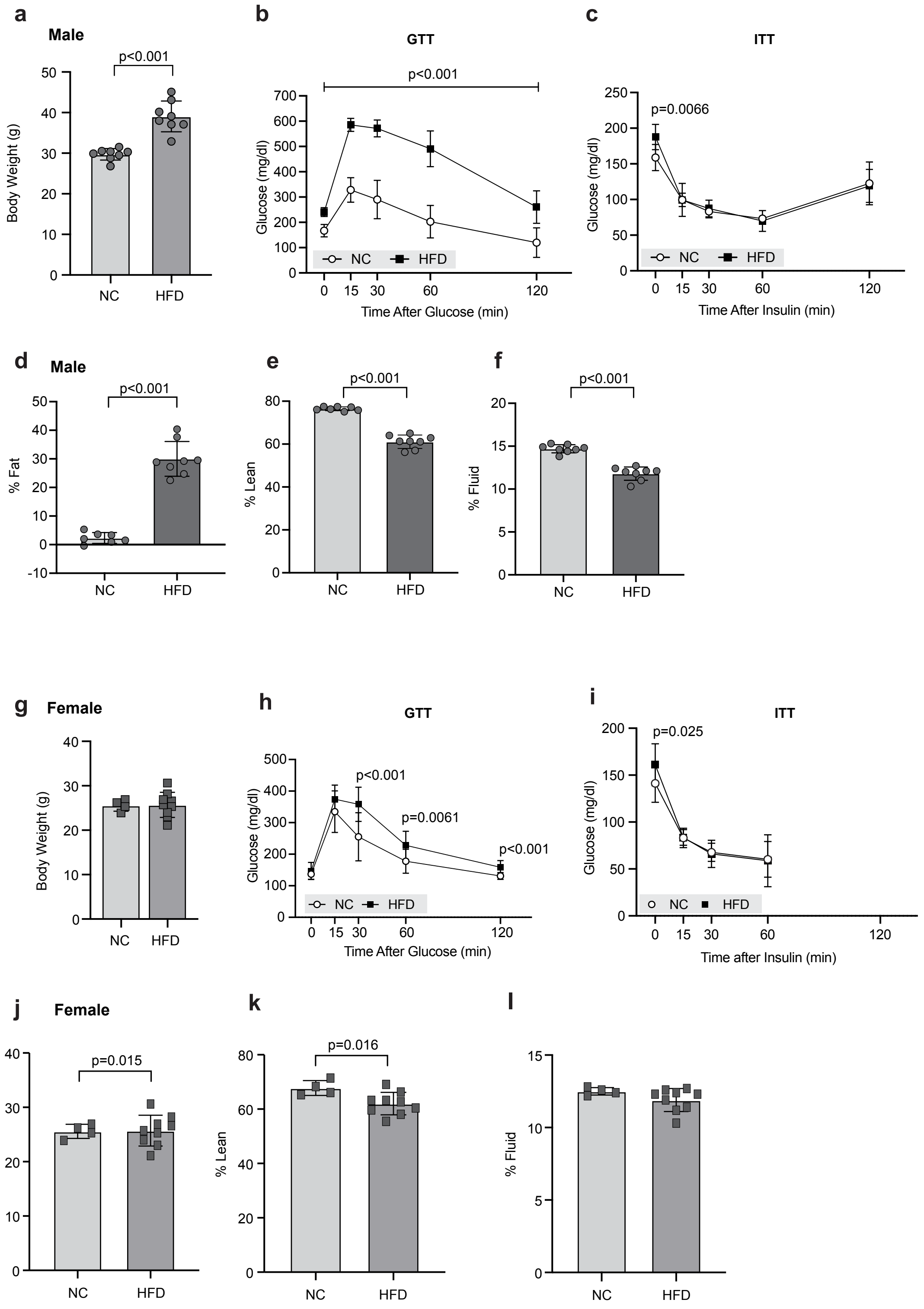
a
Male
p<0.001
Body Weight (g)
NC
HFD
b
GTT
p<0.001
Glucose (mg/dl)
NC
HFD
Time After Glucose (min)
c
ITT
p=0.0066
Glucose (mg/dl)
NC
HFD
Time After Insulin (min)
d
Male
p<0.001
% Fat
e
p<0.001
% Lean
f
p<0.001
% Fluid
g
Female
Body Weight (g)
h
GTT
p<0.001
p=0.0061
p<0.001
Glucose (mg/dl)
Time After Glucose (min)
i
ITT
p=0.025
Glucose (mg/dl)
Time after Insulin (min)
j
Female
p=0.015
% Fat
k
p=0.016
% Lean
l
% Fluid

Supplementary Figure 4: Metabolic phenotyping of male and female mice.
A and **G**. Body weight gain in male and female C57BL6/J mice after 8 weeks of normal chow (NC) or high fat diet (HFD) feeding. **B** and **H**. Glucose tolerance tests in male and female mice fed NC or HFD. **C** and **I**. Insulin tolerance tests in male and female mice fed NC or HFD. **D** and **J**. Percent (%) fat mass in male and female mice fed NC or HFD. **E** and **K**. Percent (%) lean mass in male and female mice fed NC or HFD. **F** and **L**. Percent (%) fluid in male and female mice fed NC or HFD. Values are mean ± SD.

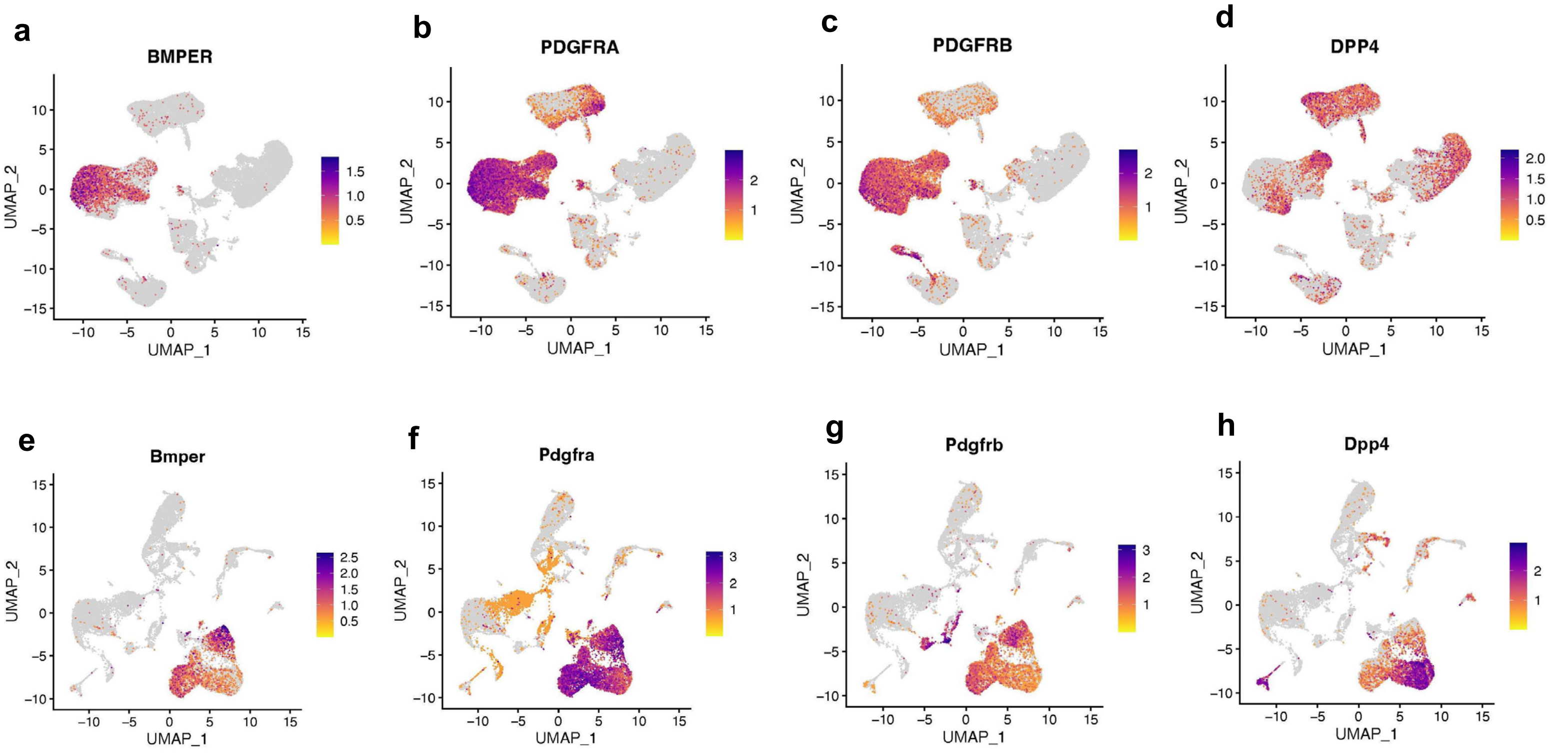
a
BMPER
b
PDGFRA
c
PDGFRB
d
DPP4
e
Bmper
f
Pdgfra
g
Pdgfrb
h
Dpp4
UMAP_1
UMAP_2

Supplementary Figure 5: UMAPs of aggregated human **(A-D)** and aggregated mouse **(E-H)** omental and perigonadal fat showing BMPER, PDGFRA, PDGFRB and DPP4 positive cells in our scRNA-seq data set. N=9 patients (2 lean male, 2 obese male, 2 lean female and 3 obese female subjects. N=4 mouse samples (1 NCD male, 1 HFD male, 1 HFD male and 1 HFD female samples with each sample including 10 mice total).

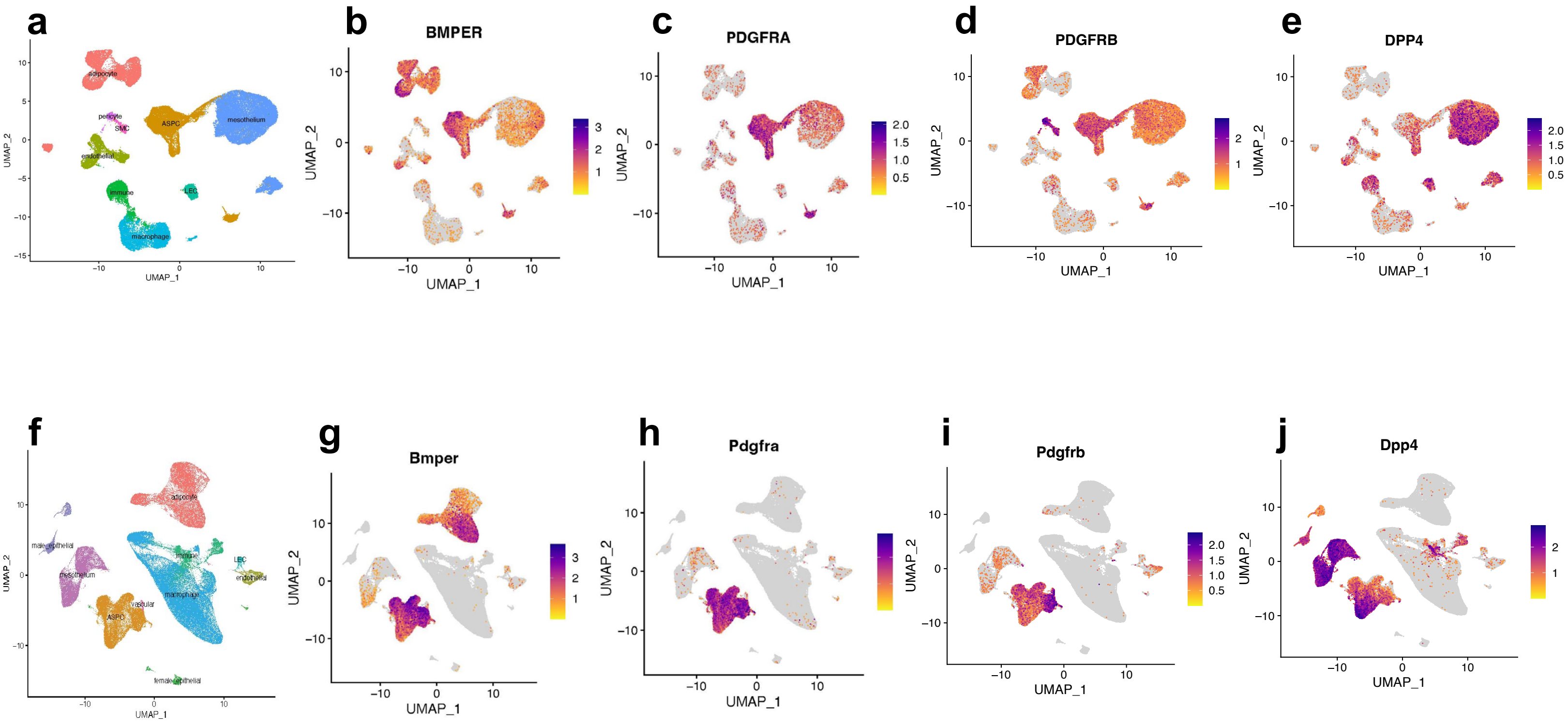

a
b
BMPER
c
PDGFRA
d
PDGFRB
e
DPP4
f
g
Bmper
h
Pdgfra
i
Pdgfrb
j
Dpp4
UMAP_1
UMAP_2
adipocyte
pericyte
SMC
ASPC
mesothelium
endothelial
immune
LEC
macrophage
male epithelial
vascular
female epithelial

Supplementary Figure 6: UMAPs of aggregated human **(A-E)** and aggregated mouse **(F-J)** omental and perigonadal fat showing BMPER, PDGFRA, PDGFRB and DPP4 positive cells in publicly available (GSE176171) single nuclei RNA sequencing (snRNA-seq). N=7 patients (1 lean male, 2 obese male, 2 lean female and 2 obese female subjects. N=14 nice (5 NCD male, 5 HFD male, 2 NCD female and 2 HFD female mice).

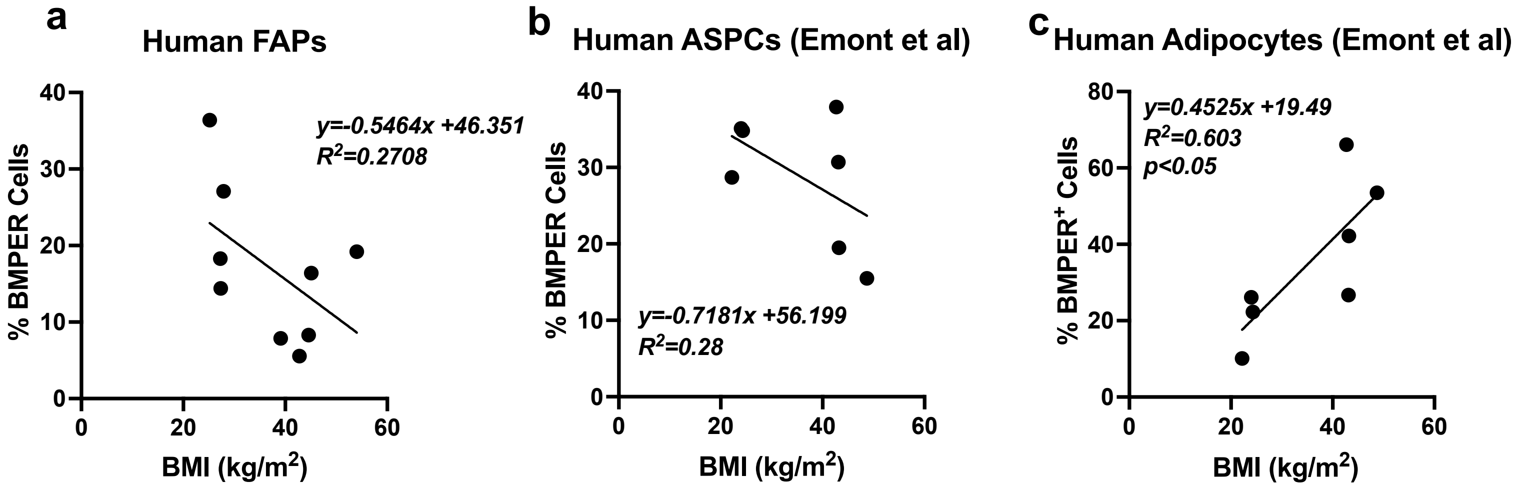
a
Human FAPs
% BMPER Cells
y=-0.5464x +46.351
R2=0.2708
BMI (kg/m2)
b
Human ASPCs (Emont et al)
% BMPER Cells
y=-0.7181x +56.199
R2=0.28
BMI (kg/m2)
c
Human Adipocytes (Emont et al)
% BMPER+ Cells
y=0.4525x +19.49
R2=0.603
p<0.05
BMI (kg/m2)

Supplementary Figure 7: Correlations between BMPER+ cells and body mass index (BMI) in human omental fat. A. Correlation between BMPER+ APCs and BMI in our study. **B** and **C**. Correlations between BMPER+ ASPCs and BMPER+ adipocytes and BMI in publicly available (GSE176171) single nuclei RNA sequencing (snRNA-seq). N=7 patients (1 lean male, 2 obese male, 2 lean female and 2 obese female subjects.

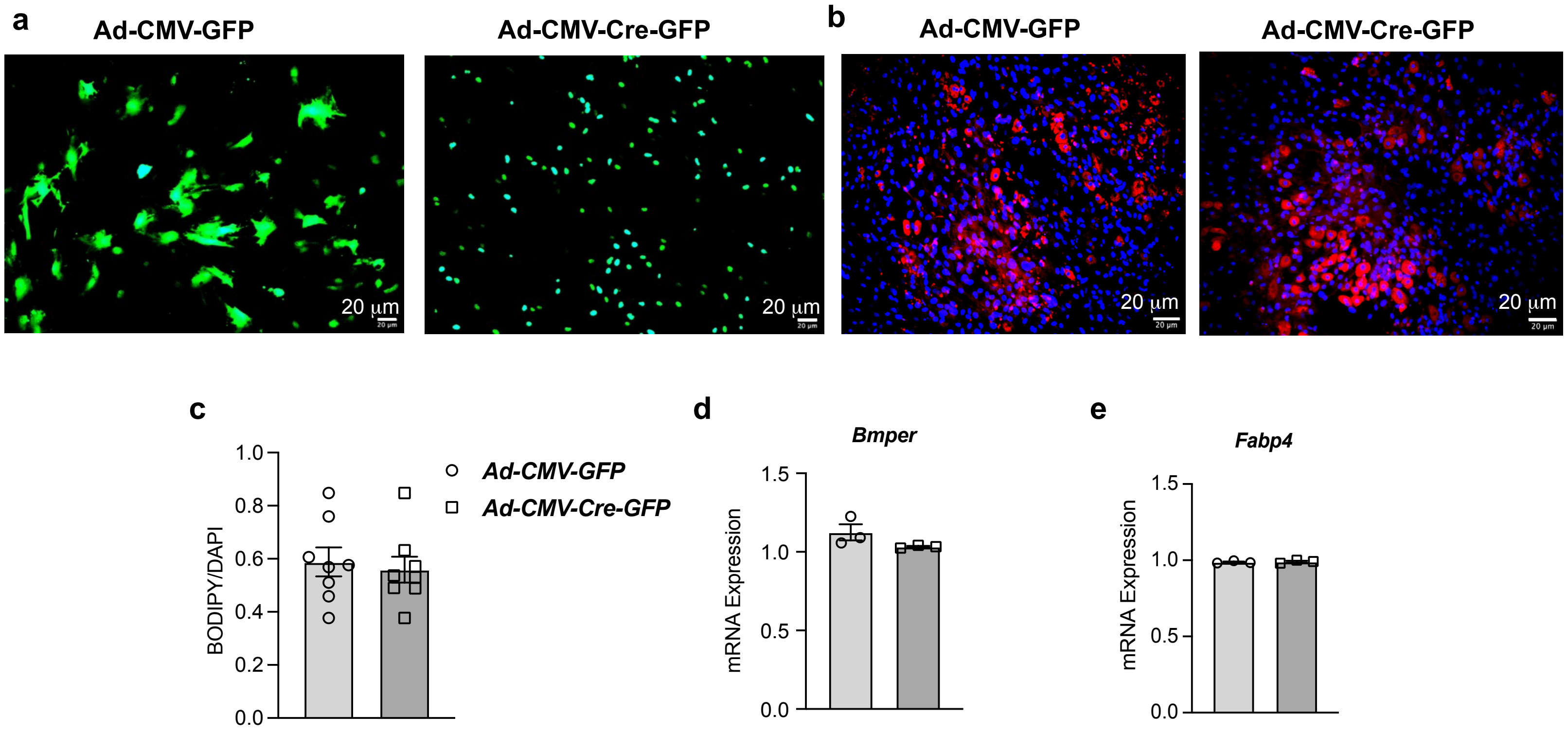
a
Ad-CMV-GFP
Ad-CMV-Cre-GFP
20 μm
20 μm
b
Ad-CMV-GFP
Ad-CMV-Cre-GFP
20 μm
20 μm
c
BODIPY/DAPI
1.0
0.8
0.6
0.4
0.2
0.0
Ad-CMV-GFP
Ad-CMV-Cre-GFP
d
Bmper
mRNA Expression
1.5
1.0
0.5
0.0
e
Fabp4
mRNA Expression
1.5
1.0
0.5
0.0

Supplementary Figure 8: Transfection with Cre in C57BL6/J control mice did not affect adipogenesis of visceral APCs.

Adipose progenitor cells (APCs) were sorted from epididymal white adipose tissue of male C57BL6/J control mice. **A**. Representative images of green fluorescent protein (GFP) expression in visceral APCs infected with adenovirus expressing GFP control (Ad-CM-GFP) or adenovirus expressing Cre-GFP (Ad-CMV-Cre-GFP) prior to differentiation. **B**. Representative images showing lipid accumulation in APCs infected with control or Cre-GFP vectors and stained with BODIPY (red) and Dapi (Blue) following differentiation. **C**. Bodipy quantification. **D-E**. Relative mRNA expression of *Bmper* and *Fabp4* in control GFP or Cre-GFP infected cells following differentiation. Values are mean ± SD.

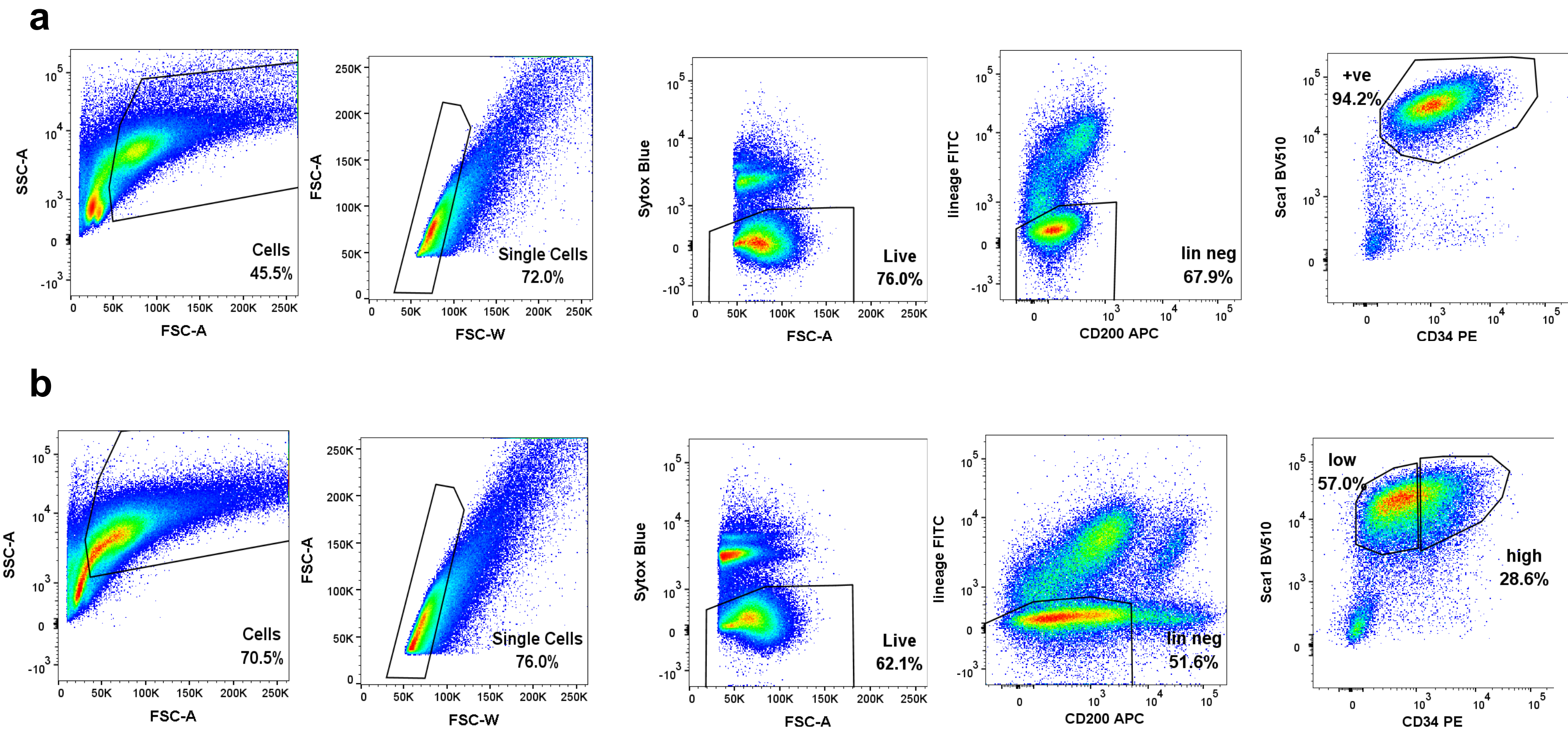
a
SSC-A
FSC-A
Cells
45.5%
FSC-A
FSC-W
Single Cells
72.0%
Sytox Blue
FSC-A
Live
76.0%
lineage FITC
CD200 APC
lin neg
67.9%
Sca1 BV510
CD34 PE
+ve
94.2%
b
SSC-A
FSC-A
Cells
70.5%
FSC-A
FSC-W
Single Cells
76.0%
Sytox Blue
FSC-A
Live
62.1%
lineage FITC
CD200 APC
lin neg
51.6%
Sca1 BV510
CD34 PE
low
57.0%
high
28.6%

Supplementary Figure 9: **Fluorescence-Activated Cell Sorting (FACS) of adipose progenitor cells.** Representative flow cytometry pseudo-color plots demonstrating FACS sorting strategy for subcutaneous **(A)** and visceral **(B)** adipose progenitor cells (APCs) in mice.

Supplementary Table 1. Human Subject Characteristics related to the publicly available single nuclei RNA sequencing data set (GSE176171) that we analyzed.

Subjects ID	Age	BMI, kg/m^2
1 Hs_OAT_01 obese Female	29	42.7
2 Hs_OAT_10 obese Female	35	43.1
3 Hs_OAT_12 obese Male	36	48.7
4 Hs_OAT_13 obese Male	24	43.2
5 Hs_OAT_254 lean Female	41	24
6 Hs_OAT_255 lean Female	73	24.3
7 Hs_OAT_266 lean Male	68	22.2
