# Supplementary material for: BMPER is a marker of adipose progenitors and adipocytes and a positive modulator of adipogenesis
Source: Commun Biol. 2023 Jun 13;6:638. doi: 10.1038/s42003-023-05011-w (PMC10264349; doi:10.1038/s42003-023-05011-w)
Supplement: Supplementary file 2 — Description of Additional Supplementary Files [file 42003_2023_5011_MOESM2_ESM.pdf]

Description of Additional Supplementary Files

File name: Supplementary Data 1

Description: Cell clusters in human stromall vascular cells isolated from omental adipose tissue

File name: Supplementary Data 2

Description: Cell clusters in mouse stromal vascular cells isolated from perigonadal adipose tissue

File name: Supplementary Data 3

Description: Cell clusters within the adipo fibro progenitors in human

File name: Supplementary Data 4

Description: Cell clusters within the adipo fibro progenitors in mouse

File name: Supplementary Data 5

Description: Pathway analysis of human cell clusters

File name: Supplementary Data 6

Description: Pathway analysis of mouse cell clusters

File name: Supplementary Data 7

Description: Source Data behind the graphs in this manuscript
