# Supplementary material for: BMPER is a marker of adipose progenitors and adipocytes and a positive modulator of adipogenesis
Source: Commun Biol. 2023 Jun 13;6:638. doi: 10.1038/s42003-023-05011-w (PMC10264349; doi:10.1038/s42003-023-05011-w)
Supplement: Supplementary file 10 — Reporting Summary [file 42003_2023_5011_MOESM10_ESM.pdf]

nature portfolio

Corresponding author(s): Sihem Boudina
COMMSBIO-22-3572A

Last updated by author(s): 3/30/2023

Reporting Summary

Nature Portfolio wishes to improve the reproducibility of the work that we publish. This form provides structure for consistency and transparency in reporting. For further information on Nature Portfolio policies, see our Editorial Policies and the Editorial Policy Checklist.

Statistics

For all statistical analyses, confirm that the following items are present in the figure legend, table legend, main text, or Methods section.

n/a	Confirmed	
☐	☒	The exact sample size (*n*) for each experimental group/condition, given as a discrete number and unit of measurement
☐	☒	A statement on whether measurements were taken from distinct samples or whether the same sample was measured repeatedly
☐	☒	The statistical test(s) used AND whether they are one- or two-sided<br>*Only common tests should be described solely by name; describe more complex techniques in the Methods section.*
☒	☐	A description of all covariates tested
☒	☐	A description of any assumptions or corrections, such as tests of normality and adjustment for multiple comparisons
☐	☒	A full description of the statistical parameters including central tendency (e.g. means) or other basic estimates (e.g. regression coefficient) AND variation (e.g. standard deviation) or associated estimates of uncertainty (e.g. confidence intervals)
☒	☐	For null hypothesis testing, the test statistic (e.g. F, t, r) with confidence intervals, effect sizes, degrees of freedom and P value noted<br>*Give P values as exact values whenever suitable.*
☒	☐	For Bayesian analysis, information on the choice of priors and Markov chain Monte Carlo settings
☒	☐	For hierarchical and complex designs, identification of the appropriate level for tests and full reporting of outcomes
☒	☐	Estimates of effect sizes (e.g. Cohen's *d*, Pearson's *r*), indicating how they were calculated

Our web collection on statistics for biologists contains articles on many of the points above.

Software and code

Policy information about availability of computer code

Data collection	single cell RNA seq data was aligned using cellranger count version 3.1.0 and then clustered
Data analysis	Signle cell RNA seq and single nuclei RNA seq were analyzed using Seurat 4.0.1, SingleR 1.10.0, ClusterProfiler 4.4.4 and other open source packages from R and BioConductor (https://cran.r-project.org/ and https://www.bioconductor.org/)

For manuscripts utilizing custom algorithms or software that are central to the research but not yet described in published literature, software must be made available to editors and reviewers. We strongly encourage code deposition in a community repository (e.g. GitHub). See the Nature Portfolio guidelines for submitting code & software for further information.

Data

Policy information about availability of data

All manuscripts must include a data availability statement. This statement should provide the following information, where applicable:

- Accession codes, unique identifiers, or web links for publicly available datasets
- A description of any restrictions on data availability
- For clinical datasets or third party data, please ensure that the statement adheres to our policy

Our single cell RNA seq data is deposited in Gene Expression Omnibus (GEO) with the accession GSE214982

Human research participants

Policy information about studies involving human research participants and Sex and Gender in Research.

Reporting on sex and gender	The present study included data from male and female human subjects and male and female mice.
Population characteristics	Age and BMI for our human subjects are included in Table 1 of the manuscript
Recruitment	The 9 patients included in the study were recruited and consented patients in accordance to the University of Utah Institutional Review Board (IRB)-approved obesity biorepository at the time of laparoscopic bariatric surgery (obesity group) or laparoscopic abdominal surgery for non-cancerous, non-infected general surgery indications (lean group). All patients were white Caucasian males and females, which represents the majority of patients referred to our clinic.
Ethics oversight	the University of Utah Institutional Review Board (IRB)

Note that full information on the approval of the study protocol must also be provided in the manuscript.

Field-specific reporting

Please select the one below that is the best fit for your research. If you are not sure, read the appropriate sections before making your selection.

☒ Life sciences ☐ Behavioural & social sciences ☐ Ecological, evolutionary & environmental sciences

For a reference copy of the document with all sections, see nature.com/documents/nr-reporting-summary-flat.pdf

Life sciences study design

All studies must disclose on these points even when the disclosure is negative.

Sample size	There was no sample size calculation performed for this study as we just started the collection of the human omental fat from bariatric and non-bariatric patients. We acknowledge that this is a limitation but we hope to make this resource available for future studies.
Data exclusions	Patients were excluded from the repository if they had active malignancy, active infection, or an inflammatory general surgical issue such as appendicitis or cholecystitis at the time of surgery
Replication	We took every step to enhance reproducibility. First, we analyzed additional single RNA seq data that was recently published by the Rosen Group (GSE176171) and show that our mouse data were confirmed in that study. We also found that BMPER was also enriched in the adipose progenitors in that data set, confirming our finding. The 3T3-L1 experiments were performed in Dr. Hilgendorff lab, whereas the Adenovirus cre infection of adipose progenitors was performed in our lab. In vitro experiments were performed at least three times to confirm the data.
Randomization	No randomization was performed.
Blinding	Blinding was not possible because of the nature of the data analysis of RNAseq and the high fat feeding or the transfection.

Reporting for specific materials, systems and methods

We require information from authors about some types of materials, experimental systems and methods used in many studies. Here, indicate whether each material, system or method listed is relevant to your study. If you are not sure if a list item applies to your research, read the appropriate section before selecting a response.

Materials & experimental systems

n/a	Involved in the study	
☐	☒	Antibodies
☒	☐	Eukaryotic cell lines
☒	☐	Palaeontology and archaeology
☐	☒	Animals and other organisms
☐	☒	Clinical data
☒	☐	Dual use research of concern

Methods

n/a	Involved in the study	
☒	☐	ChIP-seq
☐	☒	Flow cytometry
☒	☐	MRI-based neuroimaging

Antibodies

Antibodies used	Rabbit Polyclonal BMPER (Abcam; AB73900; 1:150; Lot: GR3173696-2) for ICC and Mouse Golgin-97 (Thermofisher A-21270, 1:200;

	lot: CDF4).
Validation	*Describe the validation of each primary antibody for the species and application, noting any validation statements on the manufacturer's website, relevant citations, antibody profiles in online databases, or data provided in the manuscript.*

Animals and other research organisms

Policy information about studies involving animals; ARRIVE guidelines recommended for reporting animal research, and Sex and Gender in Research

Laboratory animals	8-week-old male or female C57BL/6J mice were used.
Wild animals	N/A
Reporting on sex	We used both male and female mice as we wanted to compare the cellular composition of perigonadal fat from both sexes.
Field-collected samples	N/A
Ethics oversight	University of Utah Animal Care and Use Committee (IACUC)

Note that full information on the approval of the study protocol must also be provided in the manuscript.

Clinical data

Policy information about clinical studies

All manuscripts should comply with the ICMJEguidelines for publication of clinical research and a completedCONSORT checklist must be included with all submissions.

Clinical trial registration	N/A
Study protocol	IRB_00153325
Data collection	University of Utah Sugarhouse Clinic University of Utah Surgical Short Stay And/or via phone or telehealth system
Outcomes	Single cell analysis of omental fat from lean subjects and subjects with obesity

Flow Cytometry

Plots

Confirm that:

- [x] The axis labels state the marker and fluorochrome used (e.g. CD4-FITC).
- [x] The axis scales are clearly visible. Include numbers along axes only for bottom left plot of group (a 'group' is an analysis of identical markers).
- [x] All plots are contour plots with outliers or pseudocolor plots.
- [x] A numerical value for number of cells or percentage (with statistics) is provided.

Methodology

Sample preparation	stromal vascular fractions were prepared by collagenase digestion fron adipose tissue and were incubated in red blood cell lysis buffer for 2 minutes, suspended in HBSS, and labeled with antibodies against surface markers Lin- (CD31-, CD45- and Ther119-) CD200- Sca1+ CD34low). APCs were then separated by fluorescence-activated cell sorting (FACS) under sterile conditions.
Instrument	BD FACSARIA
Software	BD FACSDiva Version 8 Software
Cell population abundance	The adipose progenitor cells abundance varies by fat depot, sex and condition.
Gating strategy	Cells were initially selected by size, on the basis of forward scatter (FSC) and side scatter (SSC), followed by exclusion of dead cells on the basis of uptake of Cytox. Then, live cells were gated on both SSC and FSC singlets, ensuring that the staining of individual cells was analyzed. Next, the cells were separated on the basis of the cell-surface markers indicated

- [x] Tick this box to confirm that a figure exemplifying the gating strategy is provided in the Supplementary Information.
